# Template-directed self-assembly of dynamic covalent capsules with polar interiors†
†Electronic supplementary information (ESI) available. CCDC 1566200. For ESI and crystallographic data in CIF or other electronic format see DOI: 10.1039/c7sc03731g


**DOI:** 10.1039/c7sc03731g

**Published:** 2017-09-26

**Authors:** Albano Galán, Eduardo C. Escudero-Adán, Pablo Ballester

**Affiliations:** a Institute of Chemical Research of Catalonia (ICIQ) , The Barcelona Institute of Science and Technology (BIST) , Avgda. Països Catalans 16 , 43007 Tarragona , Spain . Email: pballester@iciq.es; b ICIQ X-ray Supporting Unit , Spain; c Catalan Institution for Research and Advanced Studies (ICREA) , Passeig Lluís Companys 23 , 08010 Barcelona , Spain

## Abstract

Chiral polyimine molecular capsules with polar interiors have been prepared through template covalent dynamic self-assembly. An aryl-extended tetraaldehyde calix[4]pyrrole scaffold was condensed with suitable diamines as linkers using templates for efficient self-assembly. The capsular complexes were characterized in solution, gas phase and the solid-state. Unprecedented transfer of asymmetry was observed from a chiral diamine linker to the resulting supramolecular capsular assembly.

## Abstract 2

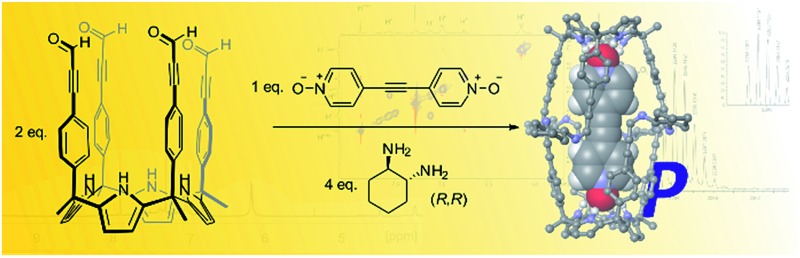
A covalent molecular capsule based on reversible imine bonds and polar interior is prepared by the template-directed self-assembly of a tetraaldehyde calix[4]pyrrole scaffold with a diamine linker.

## Introduction

Molecular encapsulation has emerged as an attractive tool in host–guest chemistry to stabilize reactive intermediates, promote or accelerate chemical transformations and even alter their typical regio- and stereo-chemical outcomes.^1^–^4^ The first molecular containers able to encapsulate neutral guests and isolate them from the bulk solution featured multiple covalent linkages between two cup-shaped hemispheres. The container's structures were rather spherical, displayed hydrophobic internal cavities and suffered from long and tedious low-yielding syntheses.^5^ They were first reported by Cram (carcerands)^6^,^7^ and Collet (cryptophanes)^8^,^9^ and produced irreversible (carceplexes) or reversible (cryptoplexes) “molecules within molecules” complexes, respectively. In both cases, constrictive binding was more important than attractive host–guest interactions to endorse these systems with slow exchange kinetics on the human and/or NMR timescales.^10^ Subsequently, synthetically more appealing, self-assembled molecular capsules relying on non-covalent interactions such as hydrogen bonds or stronger metal–ligand coordination bonds emerged.^11^–^15^ It is worth noting that the synthetic economy and high yield offered by reversible, thermodynamically controlled self-assembly processes are unparalleled.^16^ Assemblies formed by supramolecular capsules surrounding most of the surface of encapsulated guests are termed encapsulation complexes. Owing to the lack of both, sizeable portals for guest exchange and internal functionalization, the lifetimes of encapsulation complexes typically reflect the affinity of the capsular components' for each other rather than the affinity of the guest molecules for the container.^17^ During the last decade, dynamic covalent bonds have been applied for thermodynamically controlled self-assembly processes of capsules and cages.^18^–^20^ This strategy combines the strength of covalent bonds with the reversibility and selectivity of non-covalent interactions.^21^–^24^

The first examples of dynamic covalent capsules involved the condensation of 2 equiv. of a resorcin[4]arene tetraaldehyde cavitand with 4 equiv. of *m*-phenylenediamine to afford a hemicarcerand octaimine.^25^ Subsequent works of Warmuth and collaborators, also involving the use of tetraformyl resorcin[4]arene cavitands^26^–^28^ and several diamines, served to develop robust and reliable design concepts for the quantitative assembly of a variety of polyimine multi-component capsules. More recently, Rebek and co-workers studied the self-assembly of cylindrical dynamic covalent capsule hosts and their reversible binding of different guests. These capsules were also polyimines in nature and resulted from the condensation of a deep resorcin[4]arene tetraacetal cavitand with aromatic diamines.^29^ Invariably, the polyimine capsules lack polar functions in their inner cavities.^30^–^32^ ‡
‡For recent examples of polyimine cages with polar interiors see ref. 26–28. The reported containers have sizeable portals for guest exchange and are better referred as cages. This limitation dictates that their selectivity for guest encapsulation is restricted to size and shape complementarity in recognition;^33^ the lack of internal functions disfavours the binding of polar substrates.^34^ In contrast, biological receptors feature a combination of polar and non-polar groups in their binding sites, arranged to converge on their targets for optimum binding affinity and catalytic efficiency. For the closed cavities of synthetic supramolecular capsules, the incorporation of polar groups is not a trivial task.^35^–^37^

In this work, we report the template self-assembly of two dissymmetric octaimine capsular containers, **1** and **2**, featuring large polar interiors. These dynamic covalent capsules are based on the condensation reaction of 2 equiv. of the tetraaryl-extended calix[4]pyrrole **3**, each with four formyl groups at its upper rim, with 4 equiv. of 1,2-substituted aliphatic acyclic or cyclic diamines, **4** and **5**, respectively. The addition of 1 equiv. of bispyridyl-*N*-oxide derivatives, **6** or **7**, resulted in the quantitative assembly of the two capsular containers. The encapsulation complexes **6** ⊂ **1** and **6** ⊂ **2** were characterized in solution and in the gas phase through a complete set of high-resolution spectra. Notably, the octaimine complex **6** ⊂ **2** was assembled as a single diastereoisomer, and its structure was further characterized in the solid state by X-ray diffraction. We demonstrated the dynamic nature of **2** by partially exchanging the bound template **7** by its counterpart **6** through the simple addition of 1 equiv. of **6** to the preformed capsular assembly **7** ⊂ **2**. Based on the exchange kinetics, we conclude that the process takes place through a “bar-opening/bar-closing” mechanism, rather than the full disassembly of the capsule.^38^

## Results and discussion

**Scheme 1:**
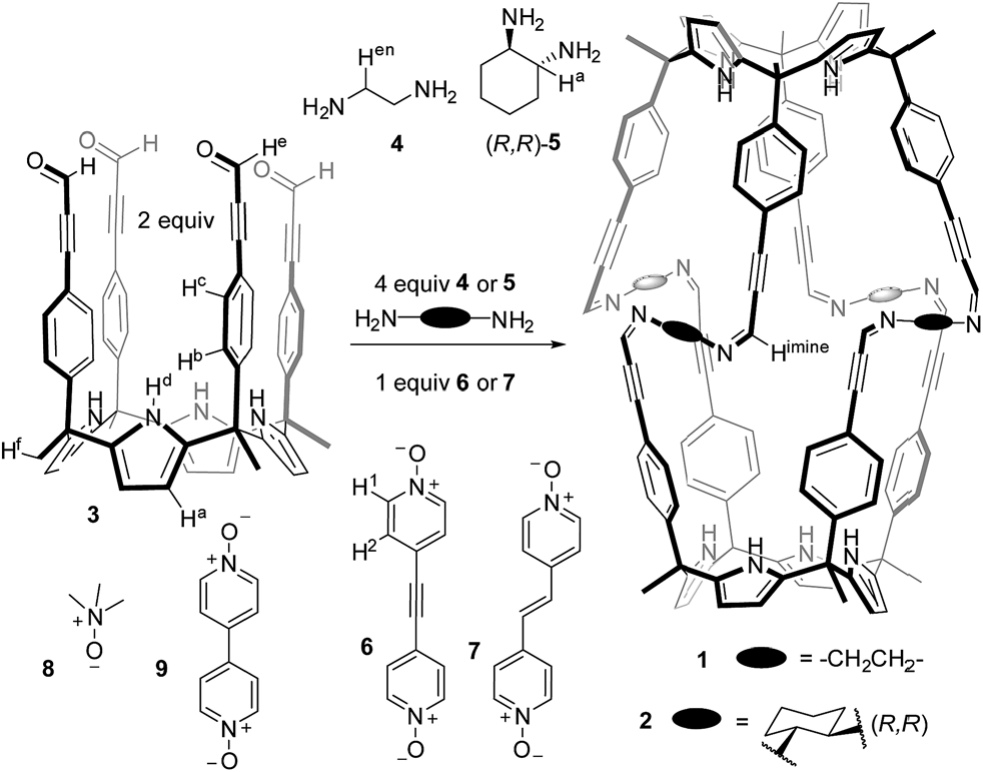
Molecular structures of octaimine capsules **1–2**, tetraaldehyde calix[4]pyrrole **3**, diamine linkers **4–5**, templates **6–7** and non-templating *N*-oxides **8–9**.

**Fig. 1:**
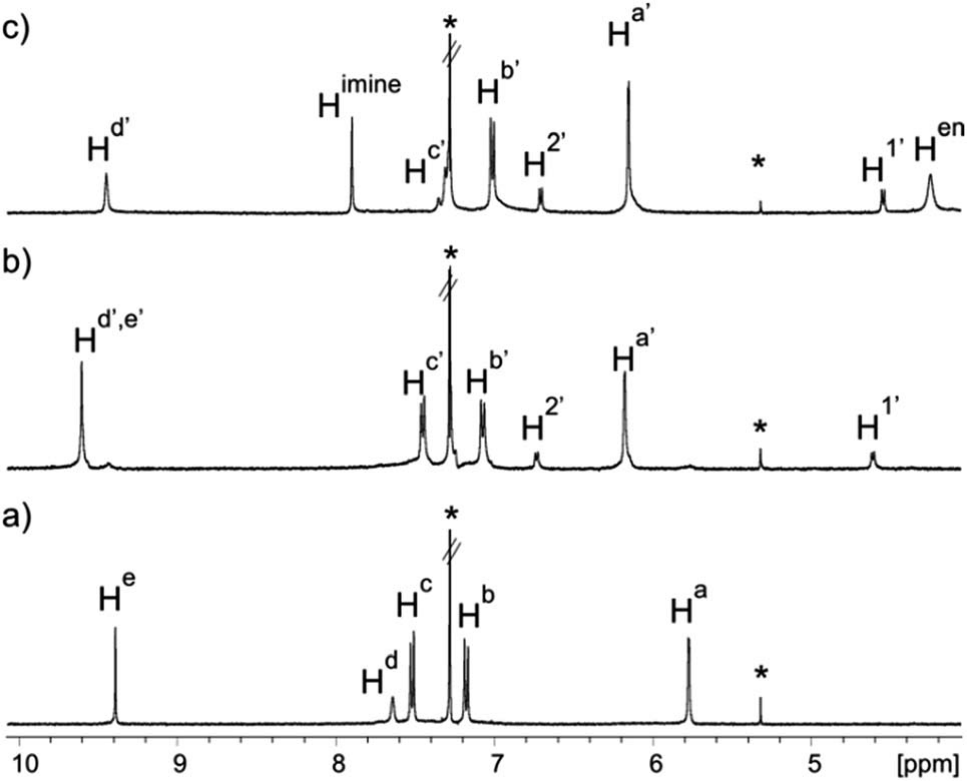
Selected regions of ^1^H NMR spectra of 1 mM CDCl_3_ solutions of: (a) calixpyrrole **3**; (b) calixpyrrole **3** and 0.5 equivalents of guest **6**; (c) calixpyrrole **3**, guest **6** and ethylenediamine in a 1 : 0.5 : 2 molar ratio (**6** ⊂ **1** complex). *Residual solvents. Primed letters and numbers indicate bound protons. See Scheme 1 for proton assignment.

Tetraaldehyde calixpyrrole **3** was prepared by Sonogashira coupling of the aryl-extended α,α,α,α-tetraiodo calixpyrrole **S1** with propargyl aldehyde diethyl acetal, followed by subsequent hydrolysis in acidic media (see ESI†). The tetraaldehyde **3** was reacted with 1,2-ethylenediamine **4** (Scheme 1) to generate **1**. The ^1^H NMR spectrum of **3** in CDCl_3_ solution showed sharp and well-defined proton signals in agreement with *C*_4v_ symmetry (Fig. 1a).§
§In CDCl_3_ solution tetraaldehyde **3** is expected to adopt an alternate conformation (1,2 or 1,3). The observation of a number of proton signals in agreement with *C*_4v_ symmetry indicated that the interconversion between alternate conformers was fast on the chemical shift timescale. The addition of two equiv. of 1,2-ethylenediamine **4** to the CDCl_3_ solution of **3** resulted in the instantaneous formation of a white precipitate.¶
¶The precipitate was insoluble in any deuterated solvent, including DMSO-*d*_6_, preventing further investigation on its composition either by NMR or MS. The ^1^H NMR spectrum of the filtered solution showed only broad proton signals indicative of an ill-defined aggregate (Fig. S2†). The addition of two equiv of 1,2-ethylenediamine **4** to a CDCl_3_ solution of the 1 : 1 inclusion complex **8** ⊂ **3** complex also produced a white precipitate and a solution containing undefined aggregates (see ESI† for details).

Based on these results, we surmised that a ditopic template might be necessary to bring together two calixpyrrole units to prevent them from being kinetically trapped in insoluble polyimine non-capsular aggregates on reacting with the diamine. Recently, we used 4,4′-bipyridine-*N*,*N*′-dioxide **9** as a ditopic guest for the quantitative assembly of 1 : 2 sandwiched complexes with calix[4]pyrrole cavitand derivatives. The process required working at mM concentration and under strict stoichiometric control.^39^ In a putative 1 : 2 complex, **9** ⊂ **3**_2_, the intramolecular reactions required for the formation of the covalent octaimine capsule would be favoured over the intermolecular counterparts, owing to an increase in the local concentrations of the reacting amine and formyl groups. Unfortunately, a 1 mM CDCl_3_ solution containing calixpyrrole **3**, bis-*N*-oxide **9** and ethylenediamine **4** in 1 : 0.5 : 2 molar ratio again produced a precipitate and the ^1^H NMR spectrum of the remaining solution displayed broad signals (Fig. S8†).

In order to rationalize this unexpected result and to investigate the possible formation of the **9** ⊂ **3**_2_ sandwiched complex, we performed variable temperature ^1^H NMR (VT-NMR) experiments using a CDCl_3_ solution containing calixpyrrole **3** and 0.5 equiv. of bis-*N*-oxide **9** (Fig. S6†). At 213 K, the ^1^H NMR spectrum displayed well-defined proton signals, which were assigned to the presence of an equimolar mixture of the 1 : 1 complex, **9** ⊂ **3**, and free **3**. In short, bis-*N*-oxide **9** is not a suitable ditopic guest to be sandwiched between two bound calix[4]pyrrole **3** units. Most likely, in the **9** ⊂ **3**_2_ assembly the steric clashes that existed between the propargylic substituents of one calix[4]pyrrole unit and the *ortho*-aromatic protons of the oppositely bound calix[4]pyrrole are responsible for its reduced thermodynamic stability.

Next, we considered the use of the longer bis-*N*-oxide **6** for the assembly of the sandwiched **6** ⊂ **3**_2_ complex.

**Fig. 2:**
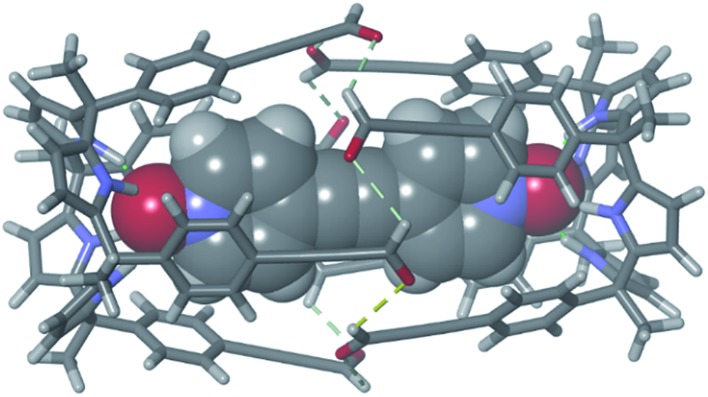
Energy-minimized (MM3) structure of the **6** ⊂ **3**_2_ capsule. Non-polar hydrogens, except formyl, in the host are removed for clarity. Included bis-*N*-oxide **6** is shown as CPK model and calix[4]pyrroles **3** are depicted in stick representation.

Simple molecular modelling studies showed that in the **6** ⊂ **3**_2_ complex the steric clashes between two 45° rotated calix[4]pyrrole units were absent (Fig. 2). Moreover, a cyclic array of eight CH···O hydrogen-bonds could be established between the unidirectionally oriented formyl groups providing additional stabilization to the assembled capsular dimer. Template **6** positions the formyl groups of the two adjacent calix[4]pyrrole hemispheres in an arrangement suitable for pairwise imine formation reactions with 1,2-ethylenediamine **4**.

The ^1^H NMR spectrum of a 1 mM CDCl_3_ solution of **3** containing 0.5 equiv. of **6** at 298 K displayed sharp signals for all protons in both binding partners (Fig. 1b). Only two highly upfield shifted doublets appeared for the aromatic protons in **6** (Δ*δ* = 3.7 and 0.7 ppm for H^1′^ and H^2′^ respectively), while the singlet of the pyrrole NHs of **3** moved downfield (Δ*δ* ∼ –2 ppm, H^d′^) in comparison to free **3**. The hydrogen atoms of the formyl groups (H^e′^) were also downfield shifted suggesting involvement in CH···O interactions. The observed number of proton signals, their chemical shift changes and the integration ratios indicated the exclusive and quantitative formation of the sandwiched **6** ⊂ **3**_2_ complex, for which we estimated a stability constant value larger than 10^8^ M^–2^. Additional support was provided by the ^1^H NMR spectrum of a 1 mM CDCl_3_ solution of **3** containing only 0.25 equiv. of **6** (Fig. S7†). To our delight, we observed two sets of separate proton signals of equal intensity, which corresponded to the protons of free tetraaldehyde **3** and its bound counterpart in the **6** ⊂ **3**_2_ complex. The two species experience slow chemical exchange on the ^1^H NMR chemical shift timescale. We calculated a diffusion constant value of 4.70 ± 0.06 × 10^–10^ m^2^ s^–1^ for the sandwich **6** ⊂ **3**_2_ complex. This value is in good agreement with those determined for structurally related capsular systems.^36^

**Scheme 2:**
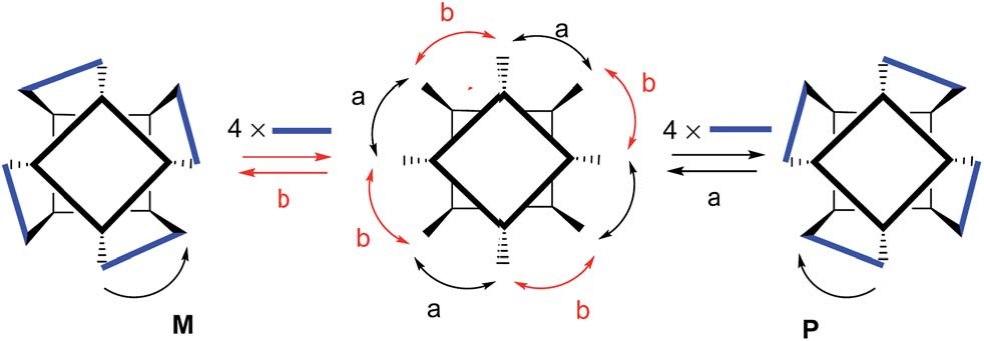
Schematic representation of the two possible senses of direction for the imine forming reactions between the eight formyl groups and the four diamine linkers. The *M*,*P* absolute configuration of the resulting capsule is assigned based on the sense of the twist (anticlockwise or clockwise) necessary to superimpose the imines groups belonging to the hemisphere facing the observer with those covalently connected.

Simple substitution of the bis-*N*-oxide **9** by its longer equivalent **6** in a 1 : 0.5 : 2 molar ratio mixture with tetraaldehyde **3** and ethylenediamine **4** produced a clear solution whose ^1^H NMR spectrum displayed sharp and nicely defined proton signals (Fig. 1c).‖
‖The use of *o*-, *m*-, or *p*-phenylenediamine as the diamine linker only produced ill-defined species, both in the presence or in the absence of a monotopic/ditopic template. Most likely, aldehyde groups in the **6** ⊂ **3**_2_ capsule are very close to each other to incorporate the aromatic linkers. The NMR signature was in complete agreement with the formation of the octaimine covalent capsule **1** trapping the bis-*N*-oxide template **6** and featuring *C*_4_ symmetry. Specifically, the signal for the aldehyde proton (H^e′^) in the **6** ⊂ **3**_2_ complex disappeared and a new singlet corresponding to the proton of the newly formed imine bonds (H^imine^) in the **6** ⊂ **1** capsule emerged at *δ* = 7.90 ppm. The methylene protons of the linkers (H^en^) resonated as one broad signal that was downfield shifted (*δ* = 4.25 ppm, Δ*δ* = –1.60 ppm) compared to the free diamine. This chemical shift change accompanies the formation of imine bonds in polyimine capsules.^26^ A ROESY experiment of a CDCl_3_ solution containing the **6** ⊂ **1** capsule revealed the existence of cross-peaks, due to through space intermolecular close-contacts, between capsule's protons, H^imine^ and H^d′^, and the aromatic protons of the encapsulated bis-*N*-oxide **6**, H^2′^ and H^1′^ (Fig. S11†). A molecular ion corresponding to the radical cation of the covalent capsular assembly [**6** ⊂ **1**]^+^˙ was detected in the gas phase using MALDI+ mass spectrometry (Fig. S26†). The capsular assembly **6** ⊂ **1** is dissymmetric and is obtained as racemic mixture of *M* and *P* enantiomers. The sense of direction of the pairwise covalent connections established by reacting adjacent formyl groups in opposed calix[4]pyrrole hemispheres with the diamine linker (Scheme 2) imposes an element of chirality to the capsular construct.

**Fig. 3:**
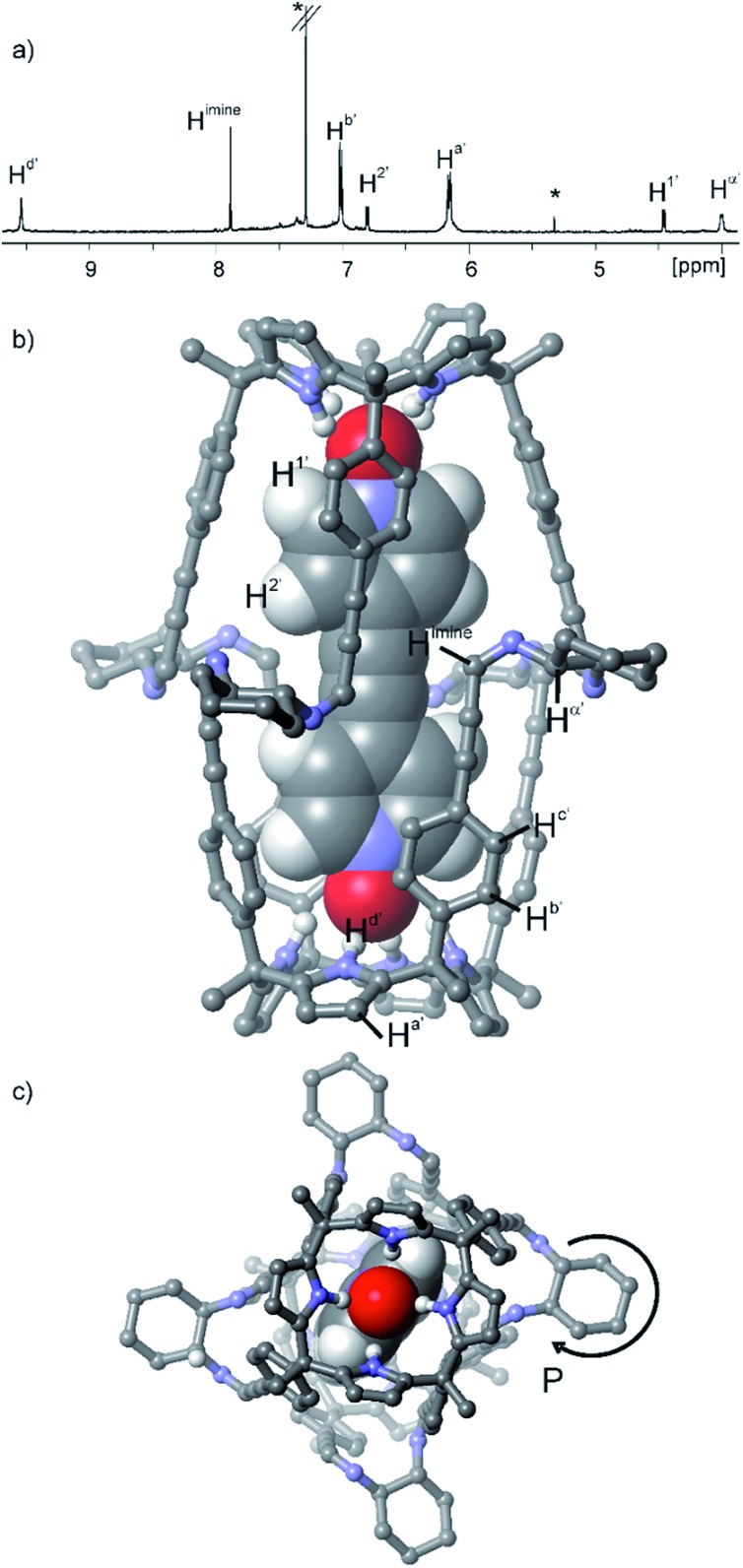
(a) Selected region of the ^1^H NMR spectrum of a 1 mM CDCl_3_ solution of calixpyrrole **3**, bis-*N*-oxide **6** and (1*R*,2*R*)-(–)-1,2-diaminocyclohexane **5** in a 1 : 0.5 : 2 molar ratio (**6** ⊂ **2** complex). *Residual solvents. Primed letters and prime numbers indicate protons in the complex. See Scheme 1 for proton assignment. (b and c) Top and side views of the X-ray structure of the **6** ⊂ **2** complex. For clarity, non-polar hydrogen atoms of capsule **2** (ball-and-stick representation) were removed. Encapsulated **6** is shown as a CPK model.

This synthetic approach could be extended to other 1,2-substituted-diamine linkers, such as the enantiomerically pure (1*R*,2*R*)-(–)-1,2-diaminocyclohexane, **5**. The addition of 2 equiv. of **5** to a 1 mM CDCl_3_ solution of the sandwich complex **6** ⊂ **3**_2_ did not produce a precipitate. The ^1^H NMR spectrum of the clear solution obtained displayed the diagnostic proton signals for the quantitative assembly of the covalent capsular complex **6** ⊂ **2** (Fig. 3a).**
**Identical NMR spectrum for the **6** ⊂ **2** complex is observed when using (1*S*,2*S*)-(+)-1,2-diaminocyclohexane. The equimolar mixture of a CDCl_3_ solution of capsule **6** ⊂ **2** formed using (–)-1,2-diaminocyclohexane and a **6** ⊂ **2** capsule formed using (+)-1,2-diaminocyclohexane did not produce any new set of signals even after days in solution. A CDCl_3_ solution of calixpyrrole **3**, guest **6** and *cis*-1,2-diaminocyclohexane in a 1 : 0.5 : 2 molar ratio only produced ill-defined species.


In the **6** ⊂ **2** complex, the imine proton resonated at 7.90 ppm, and the methyne proton, H^a′^, alpha to the nitrogen atoms of the diamine linkers, appeared downfield shifted (*δ* = 4.25 ppm, Δ*δ* = –2.7 ppm) compared to free amine (*R*,*R*)-**5**. Cross-peaks due to intermolecular close-contacts between the protons of encapsulated **6** and those of the aromatic walls and cyclohexyl linkers of capsule **2** were readily observed in a ROESY experiments (Fig. S15†). Aromatic proton peaks H^c′^ resonate as a broad hump at ≈7.4 ppm but are well-resolved at 213 K.†
†Discussion of the capsular dynamics at lower temperatures can be found in the ESI (Fig. S12–S17†). The formation of the capsular assembly **6** ⊂ **2** was also confirmed using DOSY NMR experiments that assigned a diffusion constant of 4.30 ± 0.07 × 10^–10^ m^2^ s^–1^ for its two components (Fig. S20–S25†). The cation radical [**6** ⊂ **2**]^+^˙ encapsulation complex was detected in the gas phase using high resolution MALDI+ spectrometry (Fig. S27†).

Single crystals of the **6** ⊂ **2** encapsulation complex that were suitable for X-ray diffraction analysis grew from a *p*-xylene solution (Fig. 3b). The solution of the diffraction data revealed that octaimine **2** completely surrounded the surface of the encapsulated bis-*N*-oxide **6**. The complex represents a near-perfect fit for the cavity's volume (packing coefficient = 0.53).^40^ The *N*-oxide groups of **6** formed four convergent hydrogen bonds with each one of two calix[4]pyrrole units that define the capsule's polar ends. The two molecular components also established π–π stacking and CH···π interactions. The cyclohexane rings of the amine linkers adopted a chair conformation with the amine substituents oriented in equatorial positions and experiencing *gauche* interactions. In contrast to most polyimine container molecules that feature *E*-configuration of the imine bonds, octaimine **2** displays imine bonds with *Z*-configuration. Possibly, the *Z*-configuration adds steric strain to the assembly but it provides the structural requirement for matching the dimensions of the capsule with those of the encapsulated template.

The combination of the asymmetry imposed by the unidirectional sense of the bis-imine covalent connections of the two calix[4]pyrrole hemispheres and the stereogenic carbon chirality of the enantiopure diamine linker (1*R*,2*R*)-**5**, could potentially lead to a diastereomeric mixture in the assembly of **2**: *M*-(1*R*,2*R*)-**2** and *P*-(1*R*,2*R*)-**2**. The crystal packing of the **6** ⊂ **2** complex revealed, however, the exclusively presence of the *P*-(1*R*,2*R*)-**2** diastereomer.‡
‡The NMR spectrum of capsule **2** shows exclusively one set of proton signals in agreement with the assembly of only one of the two putative diastereoisomers. This finding evidenced a strong chirality transfer from the stereogenic carbon atoms of the linker to the supramolecular chirality displayed by the assembled container. Simple molecular modelling studies (MM3) assigned a difference of *ca.* 23 kcal mol^–1^ between the two diastereoisomers *M*-(1*R*,2*R*)-**2** and *P*-(1*R*,2*R*)-**2** (see ESI†) in favor of the latter and in complete agreement with the experimental results.

Finally, we investigated the dynamic nature of the capsular container **2** by exchanging the encapsulated bis-*N*-oxide. After standing for one week in the dark, the ^1^H NMR spectrum of a solution containing the **7** ⊂ **2** complex (Fig. S18†) and one equiv. of bis-*N*-oxide **6** revealed the presence of the proton signals corresponding to the **6** ⊂ **2** encapsulation complex as a minor component of the mixture (Fig. S19†), proving the reversible nature of the capsule. The equimolar mixture of guest **7** and the **6** ⊂ **2** capsular complex did not produce the expected guest exchange even after standing in the dark for a week evidencing that the formation of complex **6** ⊂ **2** is thermodynamically favored over the formation of **7** ⊂ **2**. The similarity of our system with related polyimine hemicarcerands in combination with the slow exchange kinetics observed suggested that most likely the mechanism at operation here for guest exchange is bar-opening/bar-closing.^38^

## Conclusions

In summary, we report the templated self-assembly of tetraaldehyde calix[4]pyrrole **3** with 1,2-substituted aliphatic diamines, **4** and **5**, affording quantitatively the covalent capsules **1** and **2** stabilized by eight reversible imine bonds. The capsules feature a large polar interior in which the bis-*N*-oxides **6** and **7** used as templates are encapsulated. The resulting encapsulation complexes were characterized in solution, gas phase and the solid-state. We observed an unprecedented and efficient chirality transfer: the stereogenic carbon atoms of the diamine linker (*R*,*R*)-**5** effect the supramolecular chirality featured by the container **2**, derived from the unidirectional orientation of the reversible covalent connections. Current efforts are directed towards the reduction of the imine bonds to afford fully covalent capsules and the preparation of analogous water-soluble dynamic containers.

## Conflicts of interest

There are no conflicts to declare.

## Supplementary Material

Supplementary information

Crystal structure data
