# Supplementary material for: Template-directed self-assembly of dynamic covalent capsules with polar interiors
Source: Chem Sci. 2017 Sep 26;8(11):7746–50. doi: 10.1039/c7sc03731g (PMC5853267; doi:10.1039/c7sc03731g)
Supplement: Supplementary file 1 [file SC-008-C7SC03731G-s001.pdf]

Electronic Supplementary Material (ESI) for Chemical Science.
This journal is © The Royal Society of Chemistry 2017

Supporting Information

Template-directed self-assembly of dynamic covalent capsules with polar interiors

Albano Galán[a], Eduardo Escudero-Adán[a], Pablo Ballester*[a,b]

[a]*Institute of Chemical Research of Catalonia (ICIQ), The Barcelona Institute of Science and Technology (BIST) Avgda. Països Catalans 16, 43007, Tarragona, Spain.*

[b]*Institution for Research and Advanced Studies (ICREA), Passeig Lluís Companys, 23, 08018 Barcelona, Spain.*

pballester@iciq.es

Table of contents

1. General Methods and Instrumentation S2
2. Experimental procedures S3
3. NMR spectra S5
4. DOSY NMR experiments S21
5. Mass spectrometry experiments S27
6. Molecular modelling S29

1. General Methods and Instrumentation

Starting materials and reagents were purchased from Sigma Aldrich and used as received. All reactions were performed under argon atmosphere unless specified. Anhydrous solvents were obtained from a solvent purification system SPS-400-6 from Innovative Technologies, Inc. All solvents were of HPLC grade quality, commercially obtained and used without further purification. ^{1}H NMR and 2D NMR spectra were recorded on a Bruker Avance II 400 Ultrashield NMR spectrometer. Variable temperature experiments were performed on a Bruker Avance 500 (500.1 MHz for ^{1}H NMR) Ultrashield spectrometer. $CDCl_3$ from Sigma Aldrich was used for NMR studies. Chemical shifts are given in ppm, relative to residual solvent. Mass spectra were recorded on a LCT Premier, Waters-Micromass ESI or Autoflex, Bruker Daltonics MALDI mass spectrometer.

2. Experimental procedures

*Synthesis of Tetraaldehyde Calix[4]pyrrole **3**:*

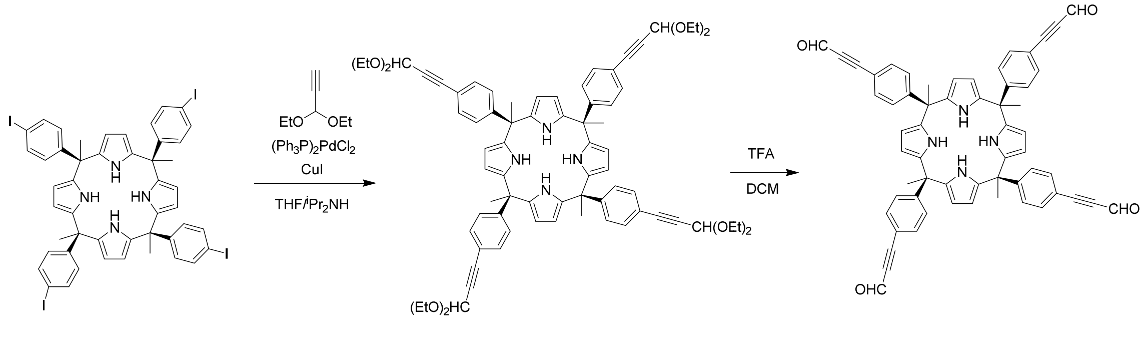

a) Tetraiodo calix[4]pyrrole[1] **S1** (300 mg, 0.256 mmol), $Pd(PPh_3)Cl_2$ (45 mg, 0.065 mmol) and CuI (24 mg, 0.128 mmol) are placed in a 50 mL Schlenk tube and dried under high vacuum for one hour. Then, a mixture of 1:1 THF and diisopropylamine (20 mL) is added under argon. Finally, propargylaldehyde diethyl acetal (293 µL, 2 mmol) is added in one portion and the reaction is stirred at room temperature under argon. After the reaction is completed as judged by TLC monitoring (ca. 1 hour) the reaction is filtered and concentrated under reduced pressure. The residue is purified by column chromatography (silica gel, dichloromethane) to obtain 300 mg of tetraacetal calixpyrrole as a bright orange solid (99% yield).

^{1}H NMR (500 MHz, $CDCl_3$) δ (ppm): 7.60 (br, 4H), 7.38 (d, J=8.4Hz, 8H), 7.08 (d, J=8.4Hz, 8H), 5.73 (br, 8H), 3.83 (dq, 8H), 3.65 (dq, 8H), 2.16 (s, 4H), 1.96 (s, 12H), 1.29 (t, J=7.2Hz, 24H). HRMS-MALDI+ m/z calcd for $C_{76}H_{84}N_4O_8$ (M^+) 1180.6289, found 1180.6256

b) Tetraacetal calixpyrrole (50 mg, 0.042 mmol) is dissolved in 40 mL of dichloromethane. Then, a solution of TFA (260 µL, 3.39 mmol) in 40 mL of dichloromethane is added **dropwise**. After the addition is completed, the reaction is washed with water (2x50 mL) and the combined organic layers are dried under reduced pressure at room temperature to obtain pure tetraaldehyde calix[4]pyrrole (34 mg, 91%). The product should be stored under argon in the fridge.

[1] Galán, A.; Aragay, G.; Ballester, P. *Chem. Sci.* **2016**, *7*, 5976

^1H NMR (500 MHz, $CDCl_3$) δ (ppm): 9.41 (s, 4H), 7.68 (br, 4H), 7.52 (d, J=8.6Hz, 8H), 7.17 (d, J=8.6Hz, 8H), 5.78 (br, 8H), 1.99 (s, 12H). HRMS-MALDI+ m/z calcd for $C_{60}H_{44}N_4O_4Na$ $(M+Na)^+$ 907.3255, found 907.3260

Experimental procedure for imine capsule formation:

In a NMR tube, 2 mg of calixpyrrole **3** are dissolved in 600 μL of $CDCl_3$. 1,2-bis(4-pyridyl)acetylene *N*,*N*'-dioxide (0.5 equivalents, 0.24 mg) are added. Once capsule formation is complete as judged by NMR analysis, diamine (ethylenediamine or (1*R*,2*R*)-(−)-1,2-diaminocyclohexane; 2 equivalents) is added. The NMR tube is agitated for a couple of minutes and then polyimine capsule is quantitatively formed. No further purification is required.

3. NMR spectra

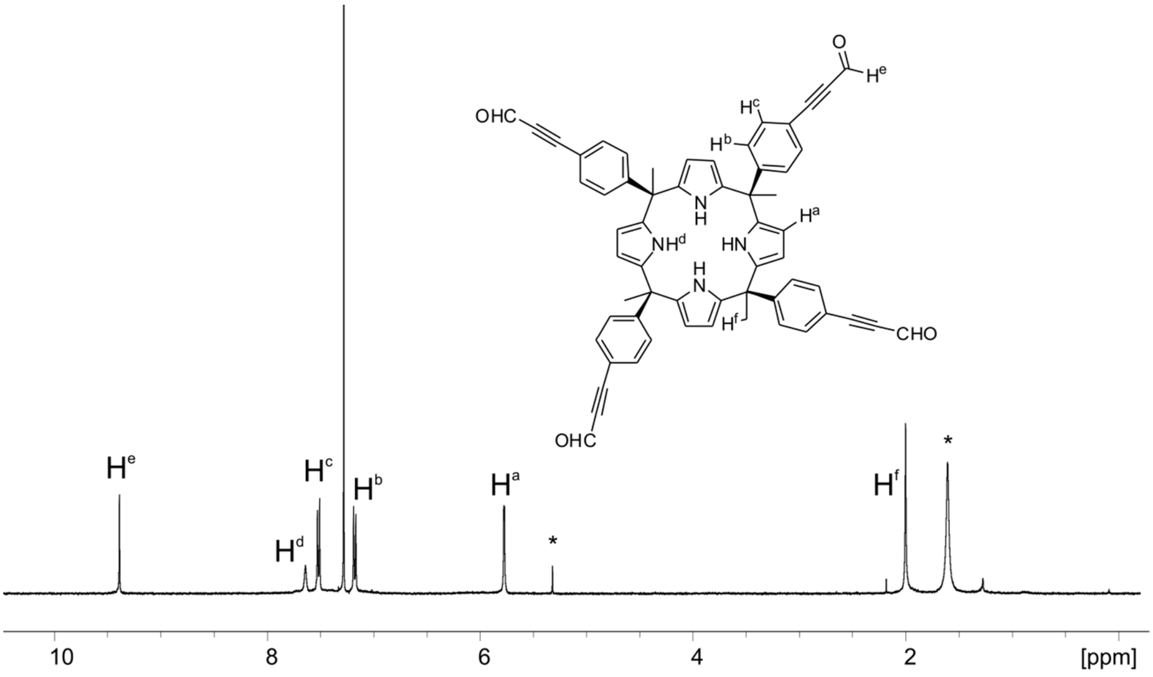

Figure S1. ^{1}H NMR spectrum of a $CDCl_3$ solution of calix[4]pyrrole **3**. *Residual solvents.

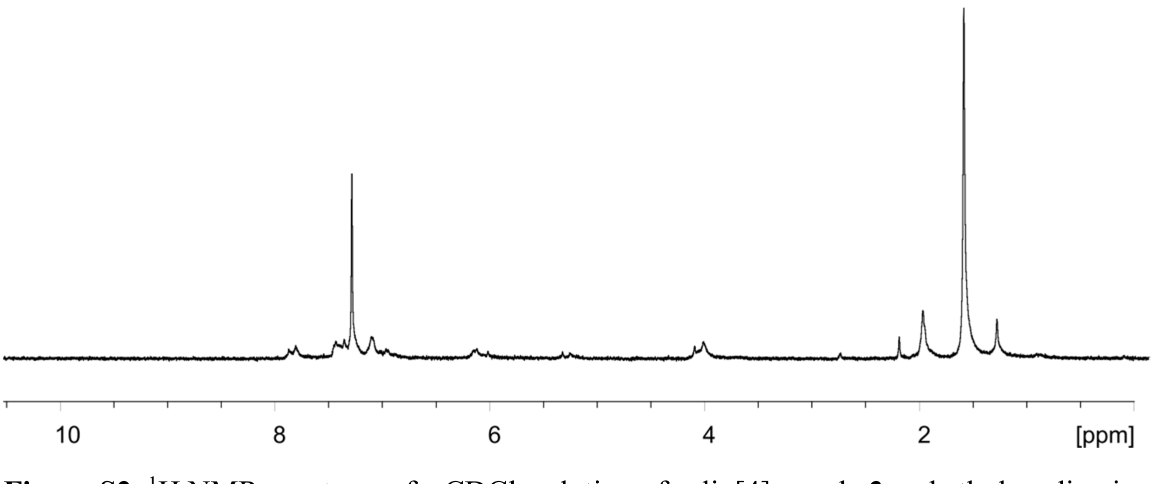

Figure S2. ^{1}H NMR spectrum of a $CDCl_3$ solution of calix[4]pyrrole **3** and ethylenediamine in a 1:2 molar ratio. *Residual solvents.

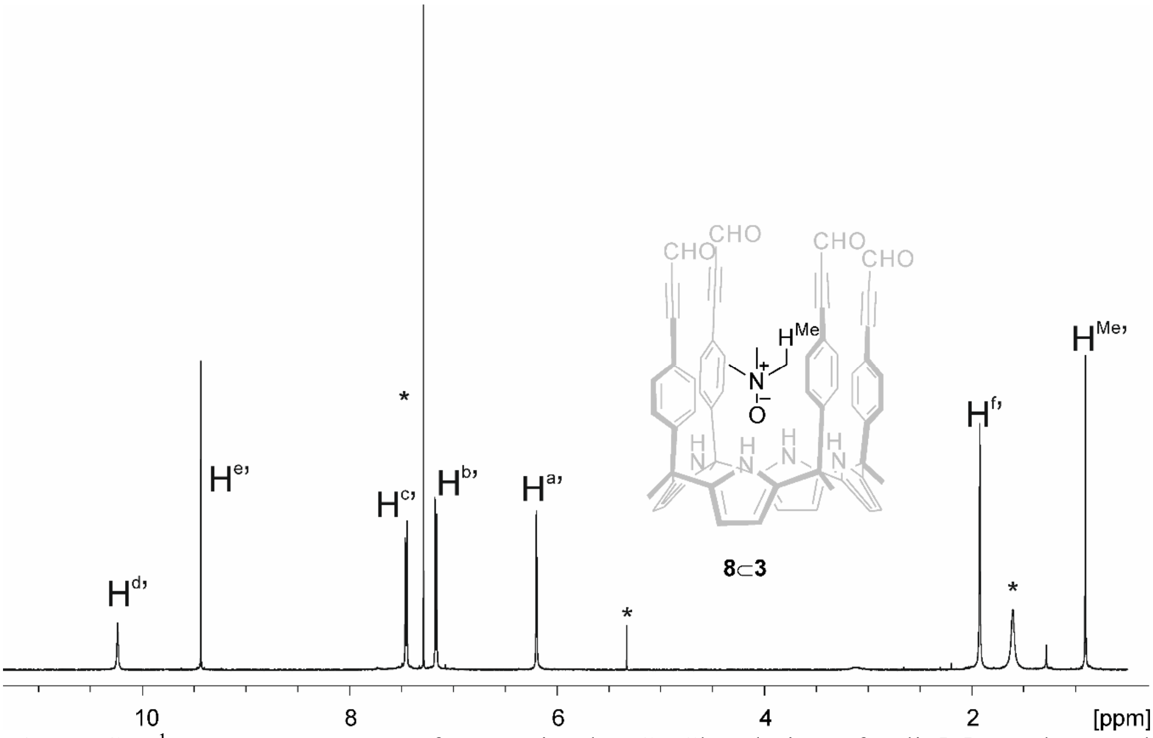

Figure S3. ^{1}H NMR spectrum of an equimolar $CDCl_3$ solution of calix[4]pyrrole **3** and trimethylamine *N*-oxide. *Residual solvents. See Figure S1 for proton assignment. Bound protons marked with primed letters and numbers.

We rationalized that the absence of a suitable template was responsible for the formation of insoluble polymeric aggregates. Aryl extended calix[4]pyrroles are known to bind amine *N*-oxides forming kinetically and thermodynamically highly stable 1:1 inclusion complexes. In these complexes, four convergent hydrogen bonds are established between the oxygen atom of the *N*-oxide group and the calixpyrrole. This process could be used to preorganize the core of **3** into the cone conformation, in which the four formyl groups are arranged in the orientation needed for the covalent assembly of the dimeric capsule. In fact, the addition of 1 equiv of trimethylamine *N*-oxide **8** to a $CDCl_3$ solution of tetraaldehyde **3** produced the diagnostic proton signals (Fig. S3) expected for the formation of the 1:1 inclusion complex, **8⊂3**. The pyrrole NHs of bound **3** (H^d, δ = 10.23 ppm; $\Delta\delta$ = -2.61 ppm) experienced a significant downfield shift. The singlet of the methyl protons for the included trimethyl *N*-oxide guest appeared upfield shifted (δ = 0.90 ppm; $\Delta\delta$ = 2.36 ppm) as a result of the strong shielding effect exerted by the four meso-aryl substituents of the host. Contrary to our expectations, the addition of two equiv of 1,2-ethylenediamine **4** to the $CDCl_3$ solution of the **8⊂3** complex also produced a white precipitate. The analysis of the solution using ^{1}H NMR spectroscopy produced complex proton signals that suggested the formation of multiple aggregated species (Fig. S4).

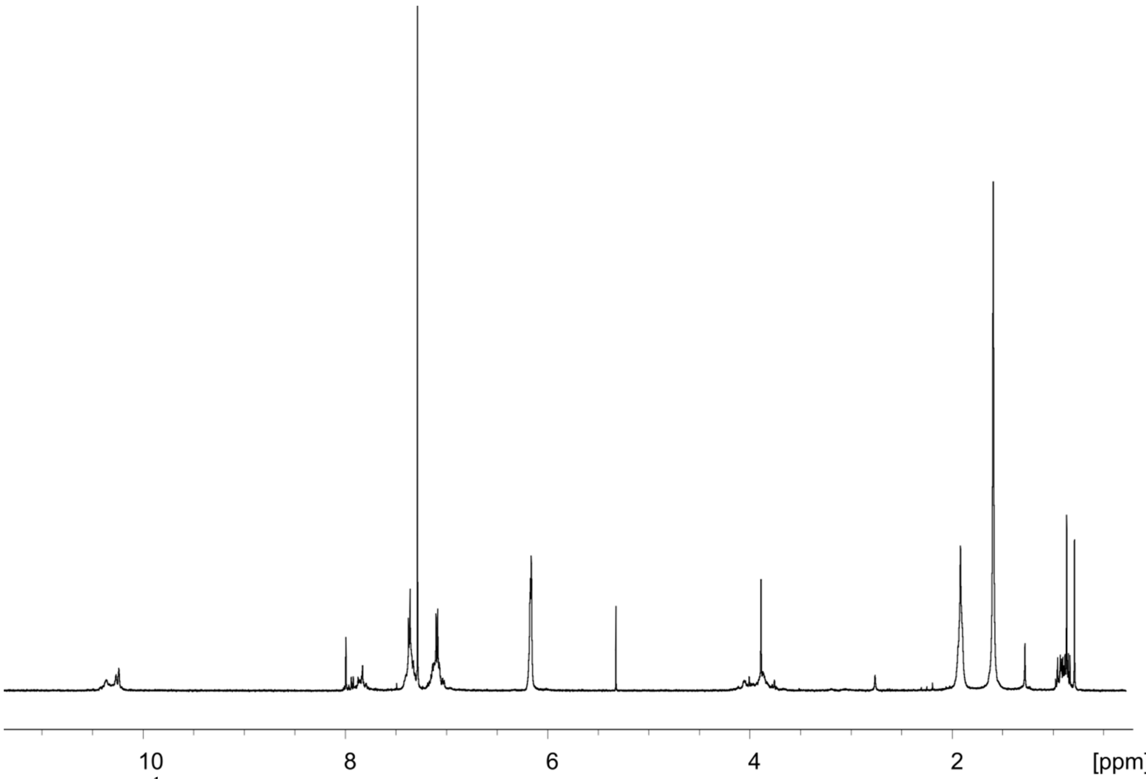

Figure S4. ^{1}H NMR spectrum of a $CDCl_3$ solution of calix[4]pyrrole **3**, trimethylamine *N*-oxide and ethylenediamine in a 1:1:2 ratio.

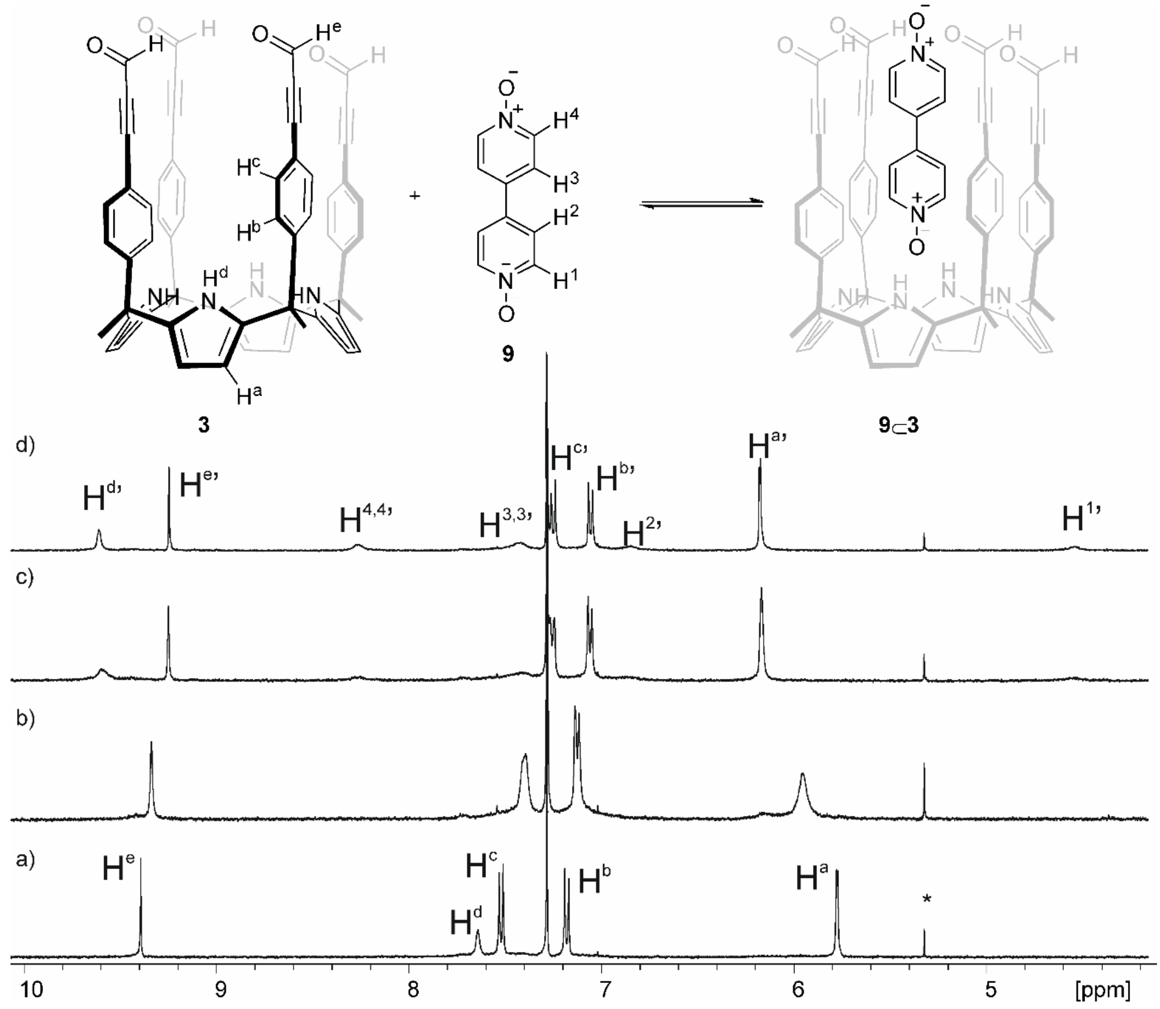

Figure S5. ^{1}H NMR titration of a $CDCl_3$ solution of calix[4]pyrrole **3** with 4,4′-dipyridyl *N*,*N*′-dioxide a) 0 eq. b) 0.5 eq. c) 1 eq. d) 1.25 eq. *Residual solvents. Protons for the 1:1 inclusion complex marked with primed letters and numbers.

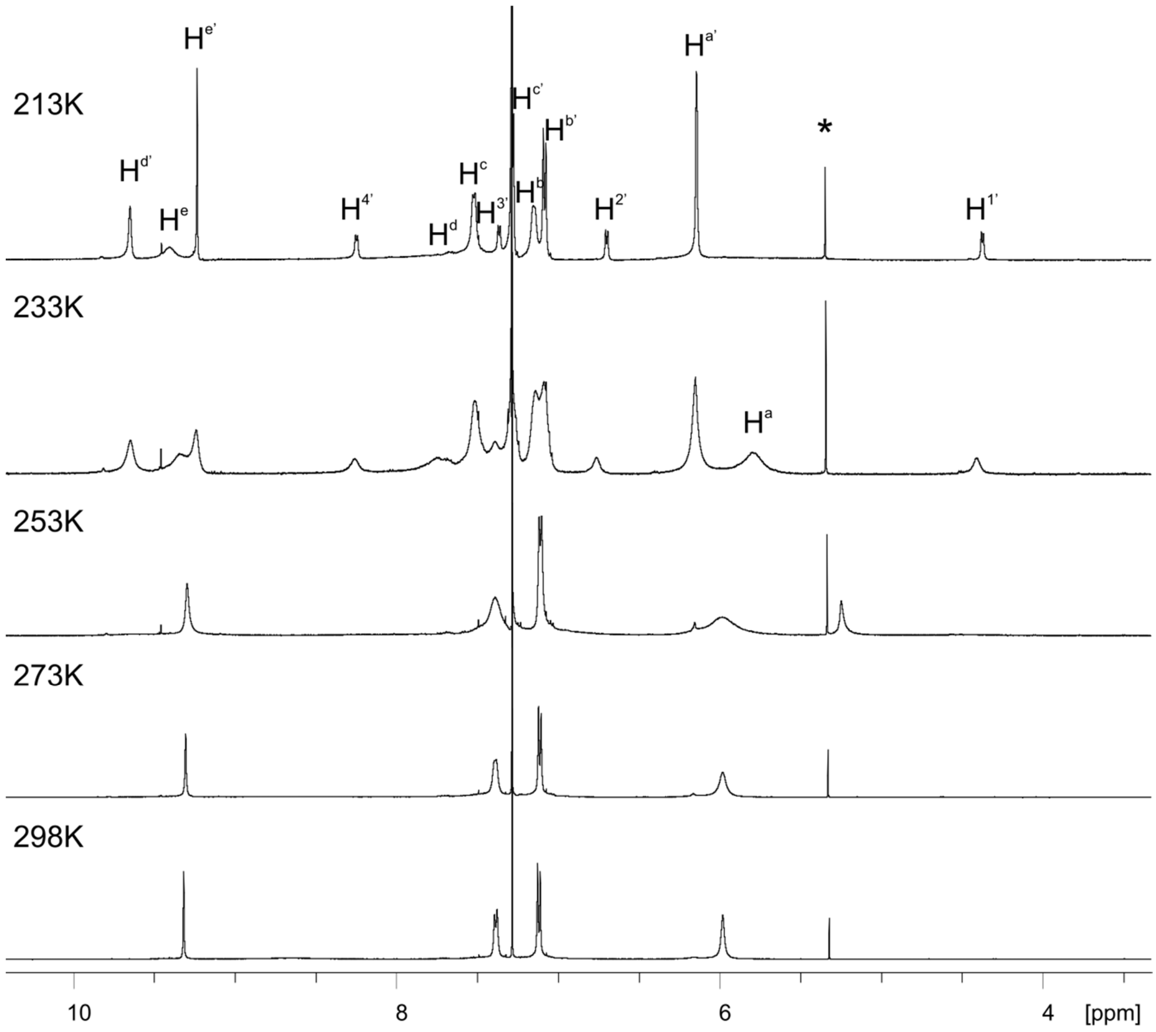

Figure S6. VT NMR spectra of a $CDCl_3$ solution of calix[4]pyrrole **3** with 0.5 equivalents of 4,4′-dipyridyl *N*,*N*′-dioxide. *Residual solvents. See Figure S1 for proton assignment. Protons for the 1:1 inclusion complex marked with primed letters and numbers.

At 298 K the ^{1}H NMR spectrum of a mixture of calix[4]pyrrole **3** and 0.5 equiv of bis-*N*-oxide **9** shows relatively sharp proton signals (Fig S6 and Figure S5b). We assigned the observed signals to the protons of calix[4]pyrrole **3** involved in a chemical exchange process between free and bound receptor (1:1 complex). Owing to the similarity in chemical shift values for the protons of **3** in the free and bound state, the exchange kinetics does not produce significant broadening to the signals. On the contrary, the signals of the protons for the guest bis-*N*-oxide **9** are broadened beyond detection. This is due to the significant changes in chemical shift values experienced by the protons in the bound guest. Specifically, the aromatic protons for the pyridyl residue of **9** included in the aromatic cavity of **3** in the 1:1 complex were upfield shifted by 3.8 ppm (*ortho* to the N atom) and 0.6 ppm (*meta* to the N-atom). For the 1:1 complex, the aromatic protons of the pyridyl residue located in the cavity defined by the triple bonds suffered reduced changes in their chemical shifts compared to free **9**. The broadening of the aromatic signals of **9** was mainly caused by a dynamic equilibria

involving the chemical exchange between the two bound pyridyl residues. We considered that although the amount of free **9** in solution was small, the chemical exchange between the two pyridyl units of bound **9** probably occurred though a decomplexation/complexation process. Figures S6 (@213 K) and S5d show the proton assignments for the 1:1 complex. The proton assignment is based on integral values, expected chemical shift values and symmetry of the complex.

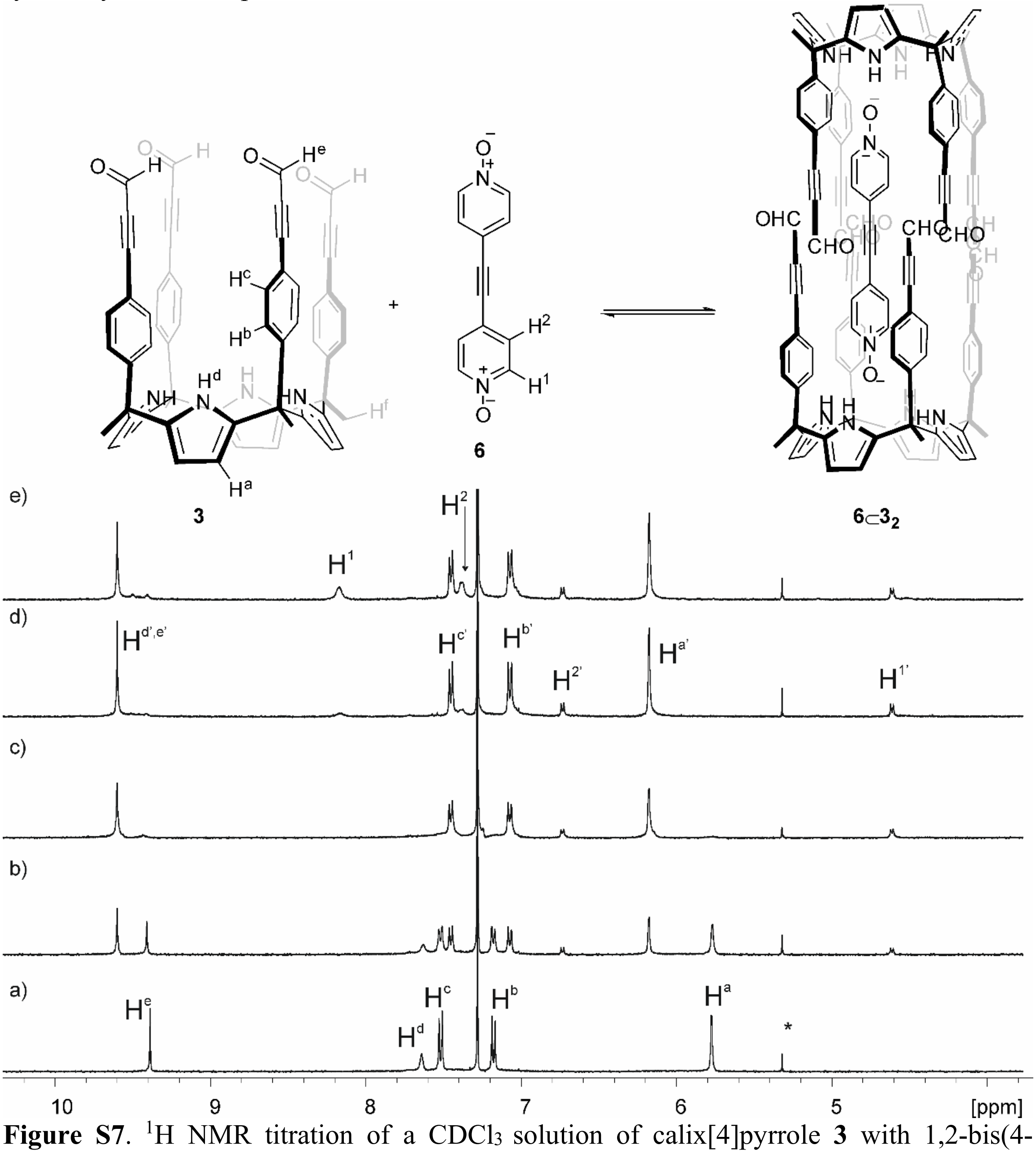

Figure S7. ^{1}H NMR titration of a CDCl3 solution of calix[4]pyrrole **3** with 1,2-bis(4-pyridyl)acetylene *N,N'*-dioxide a) 0 eq. b) 0.25 eq. c) 0.5 eq. d) 1 eq. e) 1.5 eq. *Residual solvents. See Figure S1 for proton assignment. Protons for the 2:1 capsule marked with primed letters and numbers.

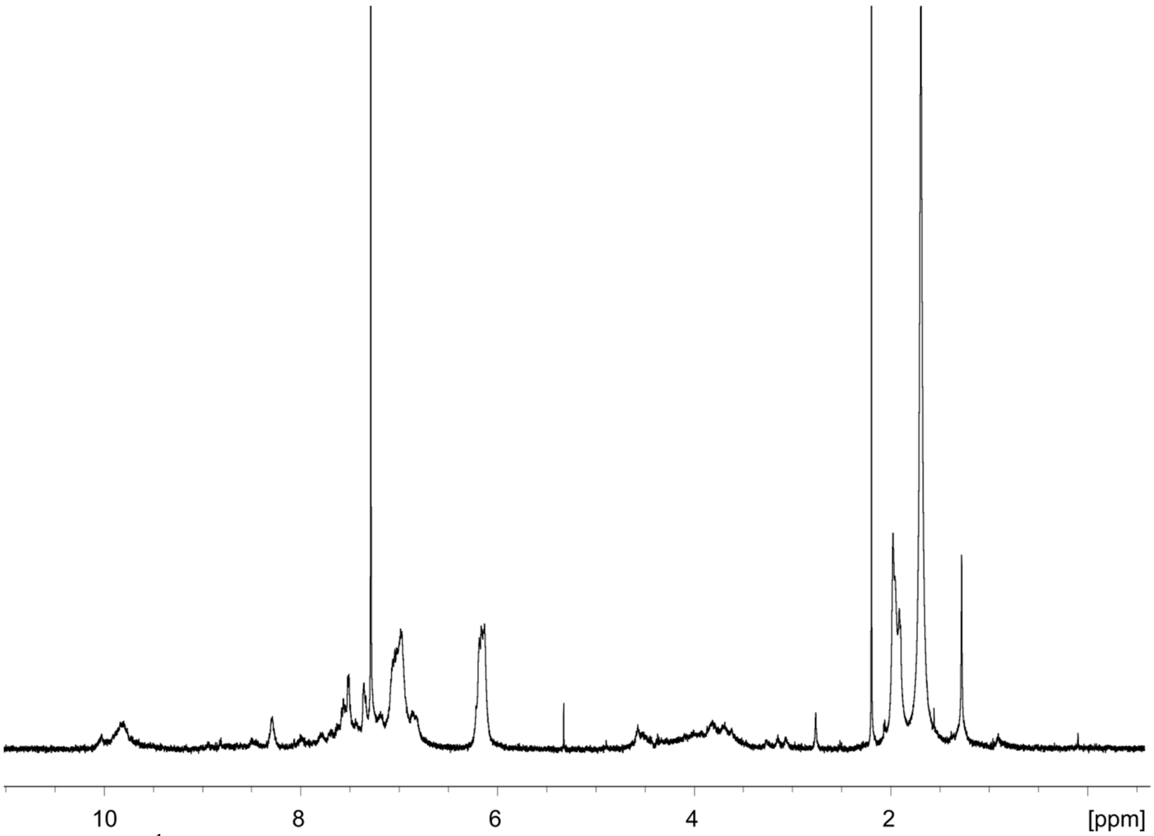

Figure S8. ^1H NMR spectrum of a $CDCl_3$ solution of calixpyrrole **3**, 4,4′-dipyridyl *N*,*N*′-dioxide and ethylenediamine in a 1:0.5:2 molar ratio.

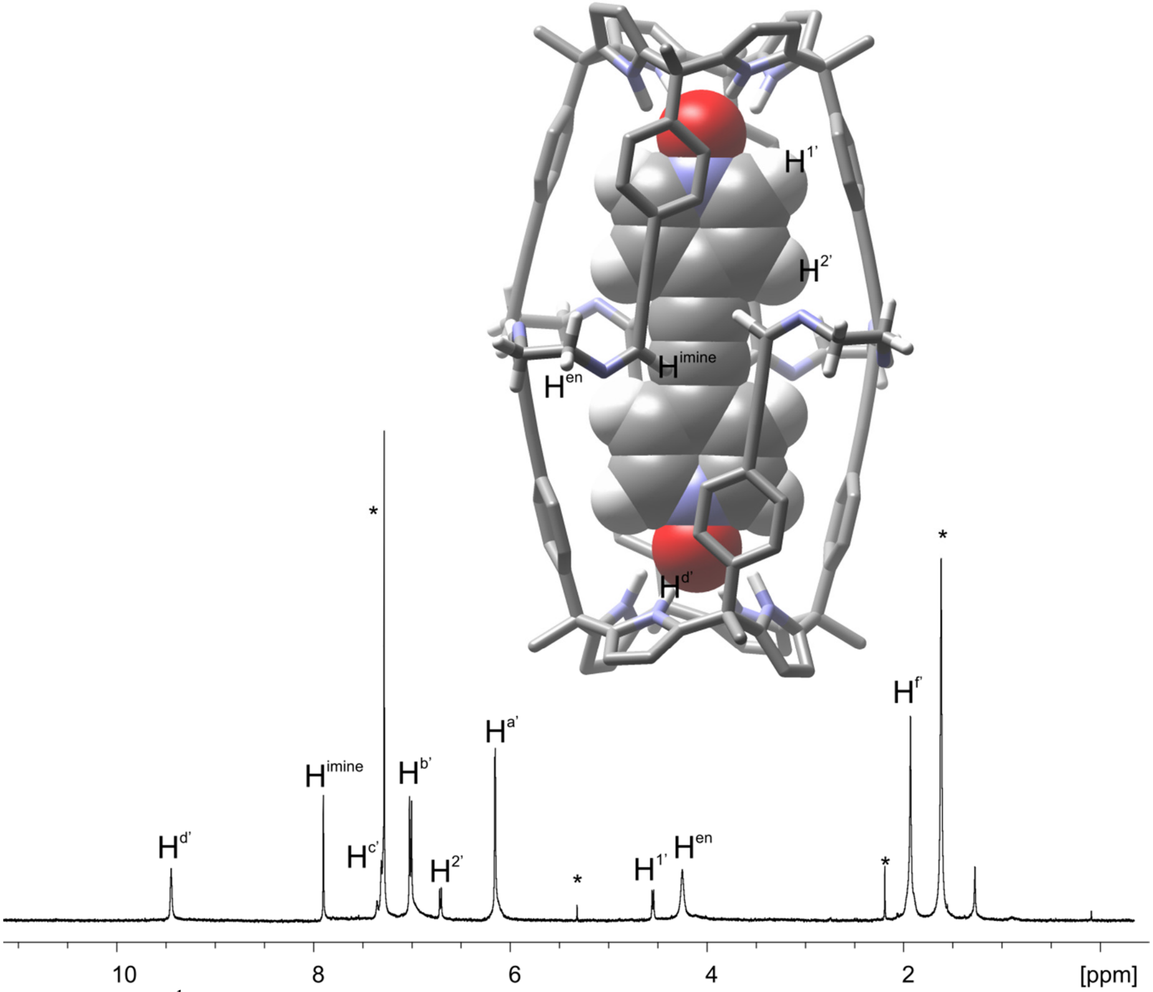

Figure S9. ^{1}H NMR spectrum of a $CDCl_3$ solution of calixpyrrole **3**, 1,2-bis(4-pyridyl)acetylene *N*,*N*'-dioxide and ethylenediamine in a 1:0.5:2 molar ratio. *Residual solvent. See Figure S1 and Figure S7 for proton assignment. Bound protons marked with primed letters and numbers. Energy-minimized (MM3) structure of the covalent capsule.

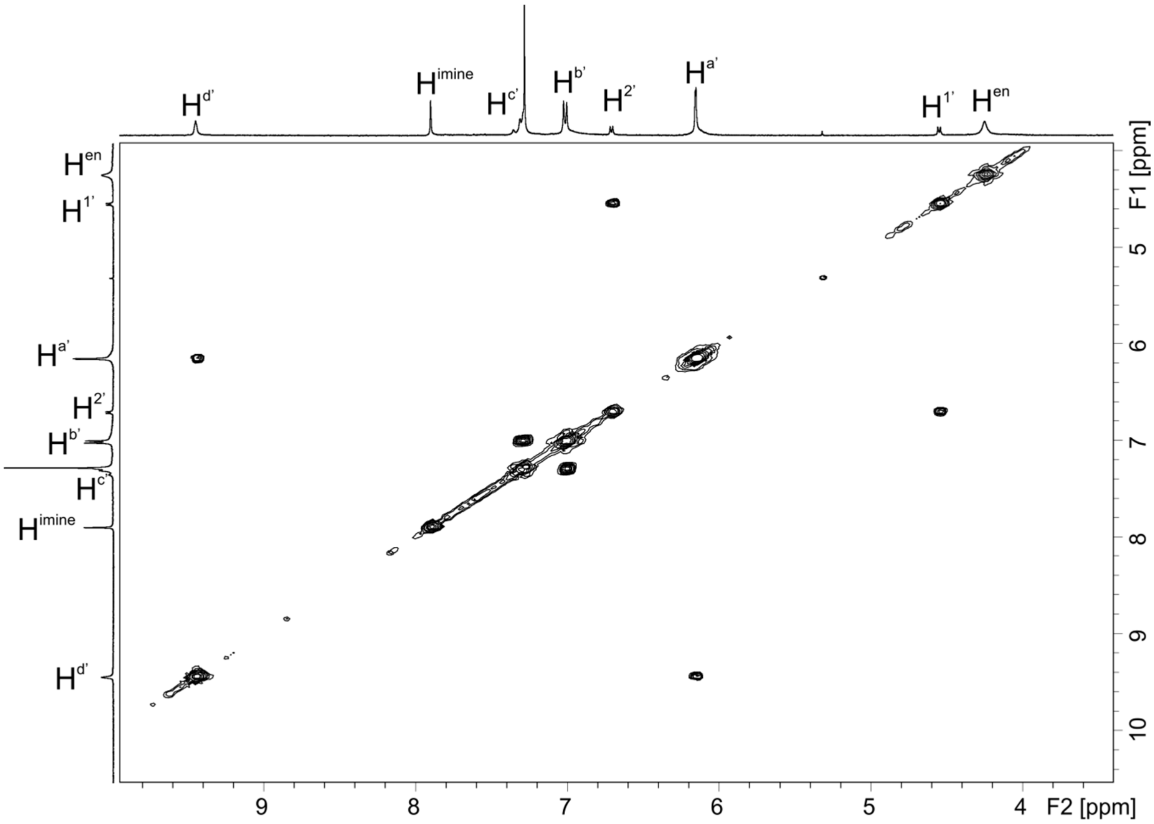

Figure S10. COSY NMR spectrum of a $CDCl_3$ solution of calixpyrrole **3**, 1,2-bis(4-pyridyl)acetylene *N*,*N*'-dioxide and ethylenediamine in a 1:0.5:2 molar ratio. See Figure S1 and Figure S7 for proton assignment. Bound protons marked with primed letters and numbers.

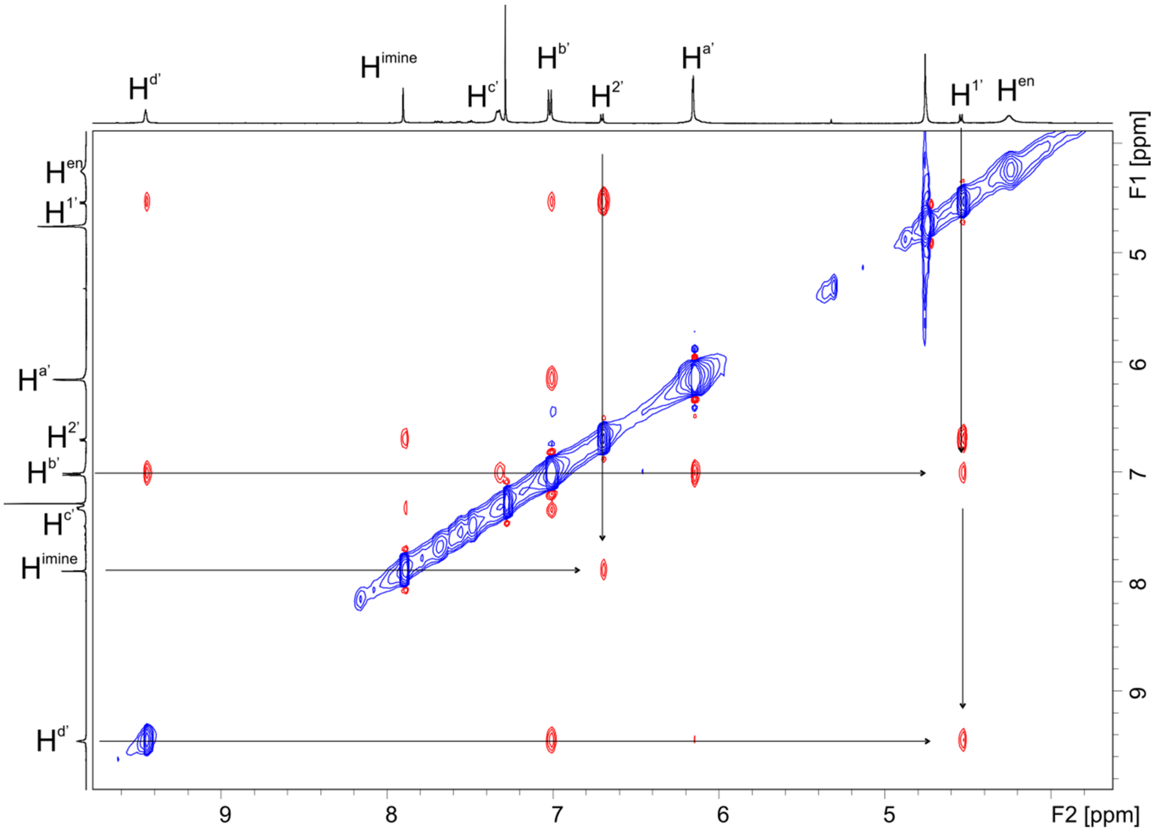

Figure S11. ROESY NMR spectrum of a $CDCl_3$ solution of calixpyrrole **3**, 1,2-bis(4-pyridyl)acetylene *N*,*N*'-dioxide and ethylenediamine in a 1:0.5:2 molar ratio. See Figure S1 and Figure S7 for proton assignment. Bound protons marked with primed letters and numbers.

We performed VT-NMR experiments using 1 mM $CDCl_3$ solutions of **6⊂1** and **6⊂2** (Figs. S12 and S16, respectively). For the **6⊂1** complex, at 213 K, we observed four separate doublets for the aromatic protons, $H^{b'}$ and $H^{c'}$, of the *meso*-aryl substituents. The $H^{c'}$ protons broadened beyond detection at room temperature. Moreover, at 213 K, the β-pyrrolic, $H^{a'}$and the methylene protons of the ethylene linker, H^{en}, resonated as diastereotopic signals. At room temperature the rotation around the $C_{\text{meso-aryl}}$ bond and the conformational motion of the ethylene spacer are fast on the NMR time scale producing the broadening and coalescence of the corresponding proton signals.

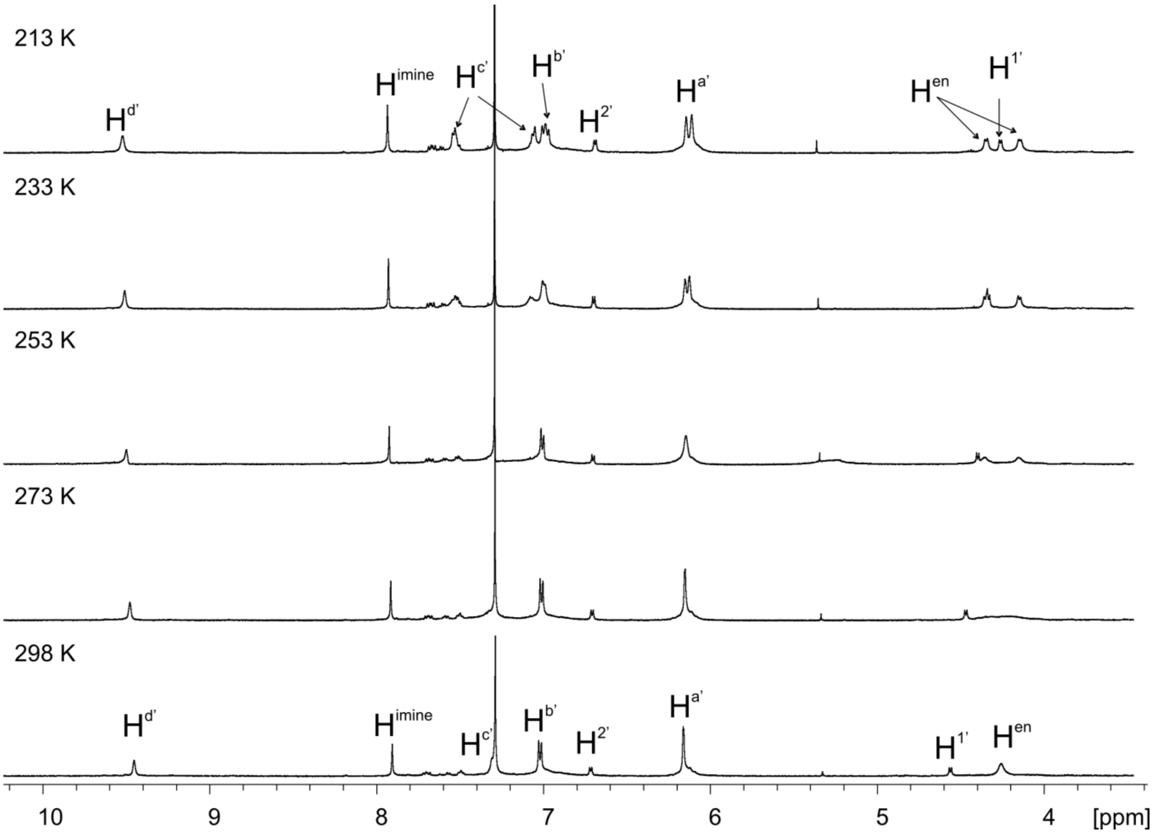

Figure S12. Variable temperature ^{1}H NMR spectra of a $CDCl_3$ solution of calixpyrrole **3**, 1,2-bis(4-pyridyl)acetylene *N*,*N*'-dioxide and ethylenediamine in a 1:0.5:2 molar ratio. See Figure S1 and Figure S7 for proton assignment. Bound protons marked with primed letters and numbers.

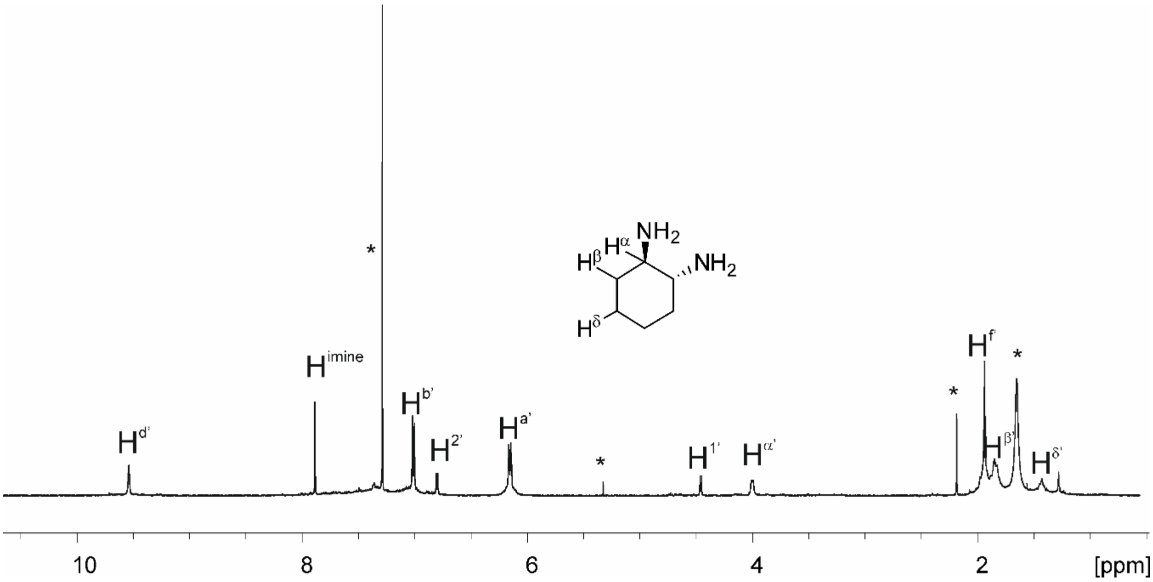

Figure S13. ^{1}H NMR spectrum of a $CDCl_3$ solution of calixpyrrole **3**, 1,2-bis(4-pyridyl)acetylene *N*,*N*'-dioxide and (1*R*,2*R*)-(−)-1,2-diaminocyclohexane in a 1:0.5:2 molar ratio. *Residual solvent. See Figure S1 for proton assignment. Bound protons marked with primed letters and numbers.

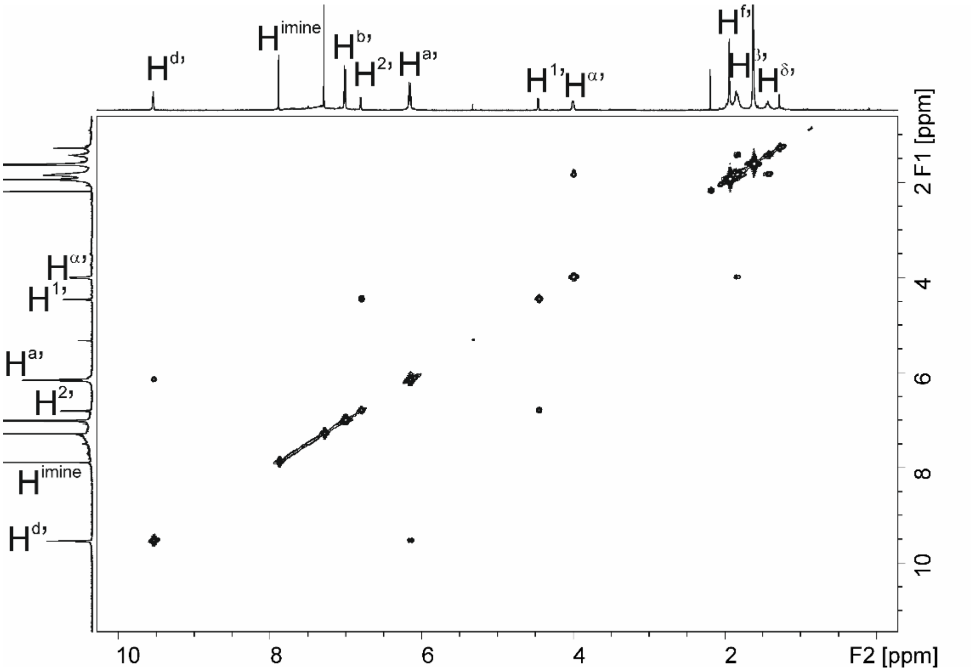

Figure S14. COSY NMR spectrum of a $CDCl_3$ solution of calixpyrrole **3**, 1,2-bis(4-pyridyl)acetylene *N*,*N*'-dioxide and (1*R*,2*R*)-(−)-1,2-diaminocyclohexane in a 1:0.5:2 molar ratio. See Figure S1, Figure S7 and Figure S13 for proton assignment. Bound protons marked with primed letters and numbers.

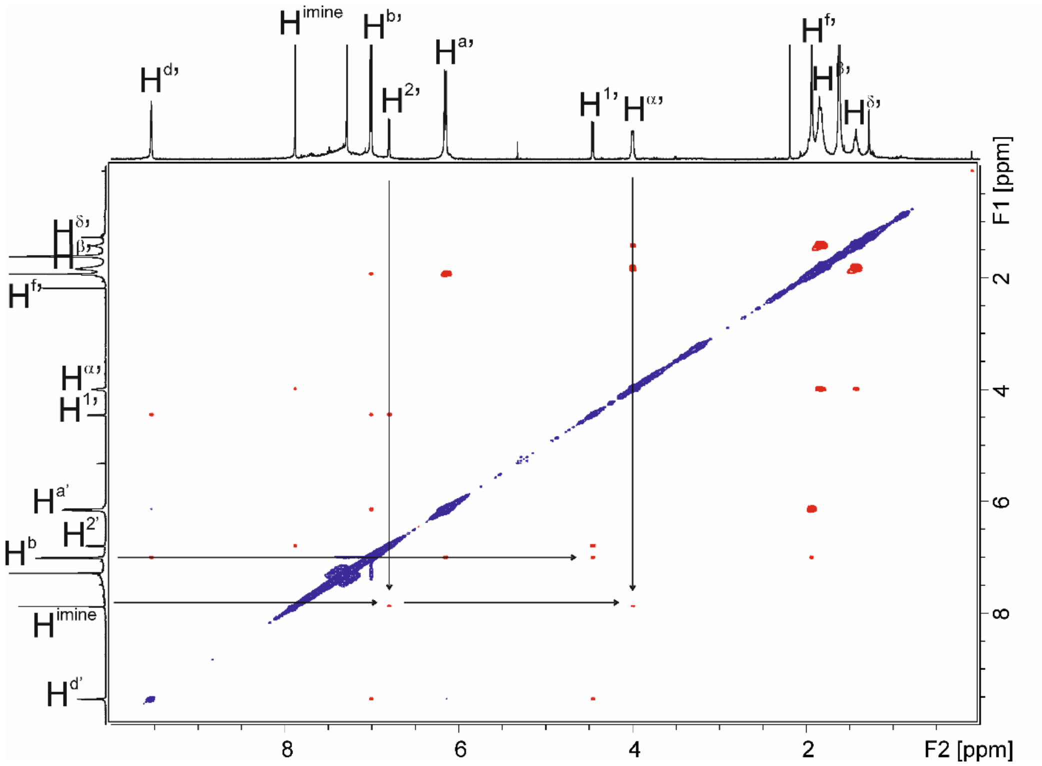

Figure S15. ROESY NMR spectrum of a $CDCl_3$ solution of calixpyrrole **3**, 1,2-bis(4-pyridyl)acetylene *N*,*N*'-dioxide and (1*R*,2*R*)-(−)-1,2-diaminocyclohexane in a 1:0.5:2 molar ratio. See Figure S1, Figure S7 and Figure S13 for proton assignment. Bound protons marked with primed letters and numbers.

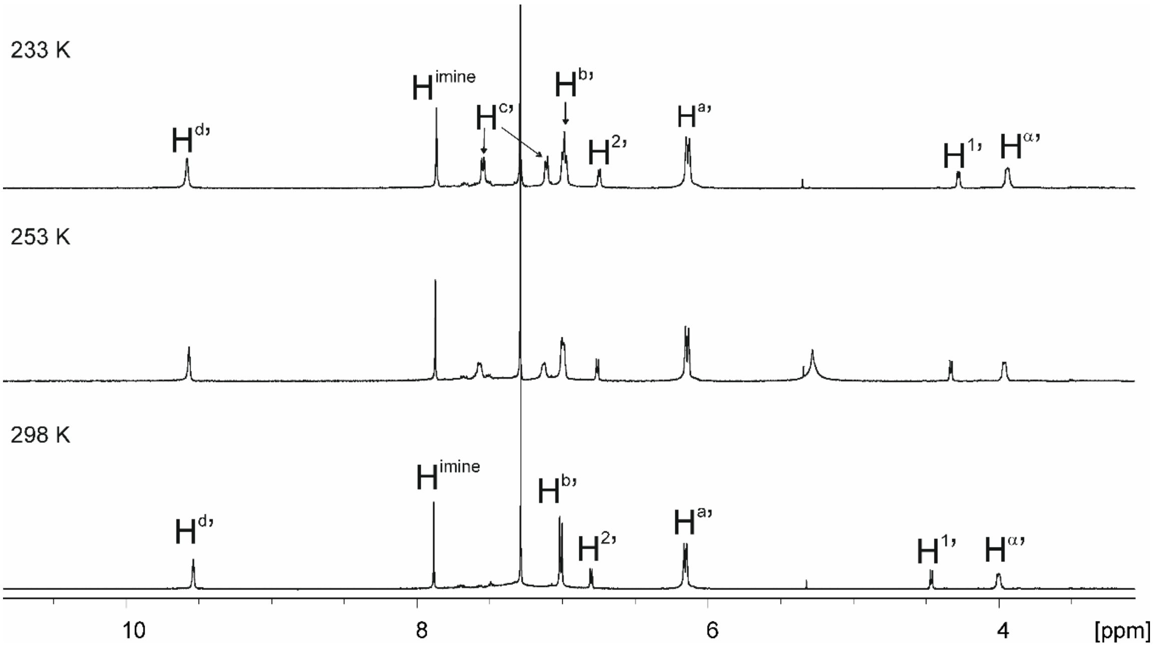

Figure S16. Variable temperature ^{1}H NMR spectra of a $CDCl_3$ solution of calixpyrrole **3**, 1,2-bis(4-pyridyl)acetylene *N*,*N*'-dioxide and (1*R*,2*R*)-(−)-1,2-diaminocyclohexane in a

1:0.5:2 molar ratio. See Figure S1, Figure S7 and Figure S13 for proton assignment. Bound protons marked with primed letters and numbers.

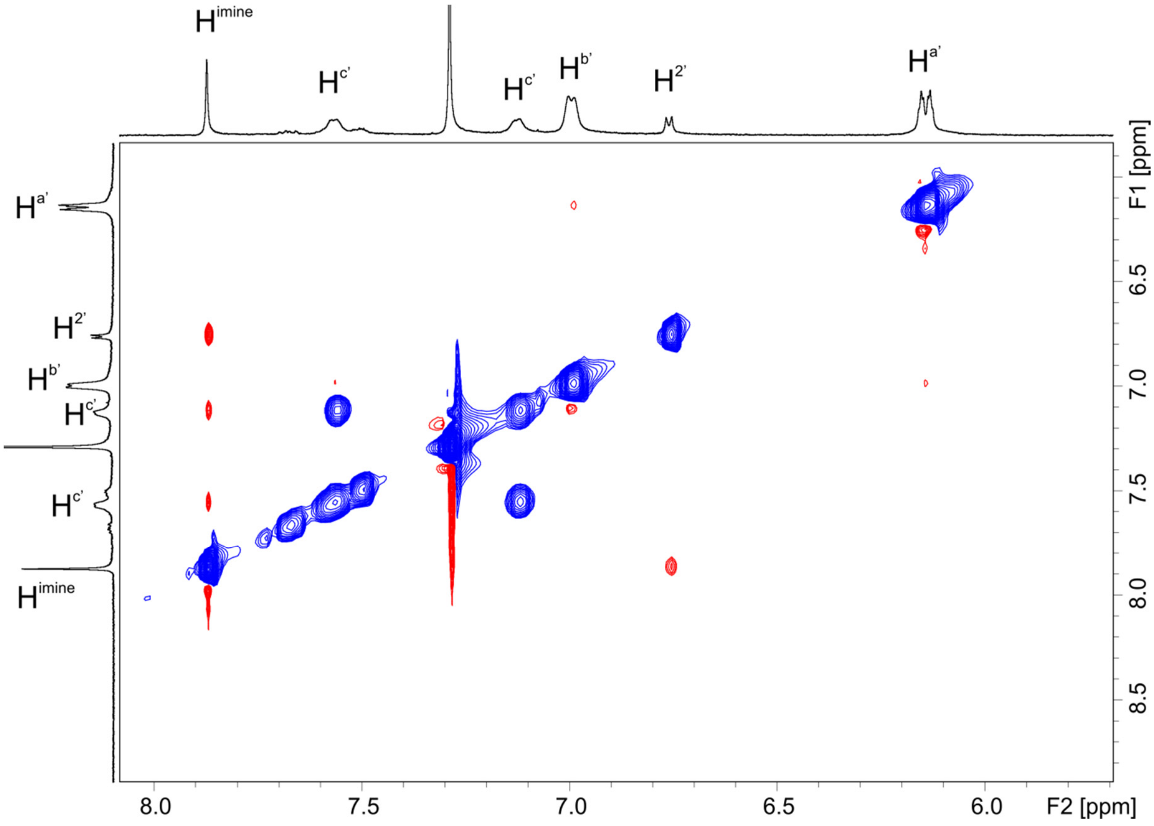

Figure S17. Selected region of a ROESY NMR spectrum of a $CDCl_3$ solution of calixpyrrole **3**, 1,2-bis(4-pyridyl)acetylene *N*,*N*'-dioxide and (1*R*,2*R*)-(−)-1,2-diaminocyclohexane in a 1:0.5:2 molar ratio performed at 253 K. See Figure S1 and Figure S7 for proton assignment. Bound protons marked with primed letters and numbers.

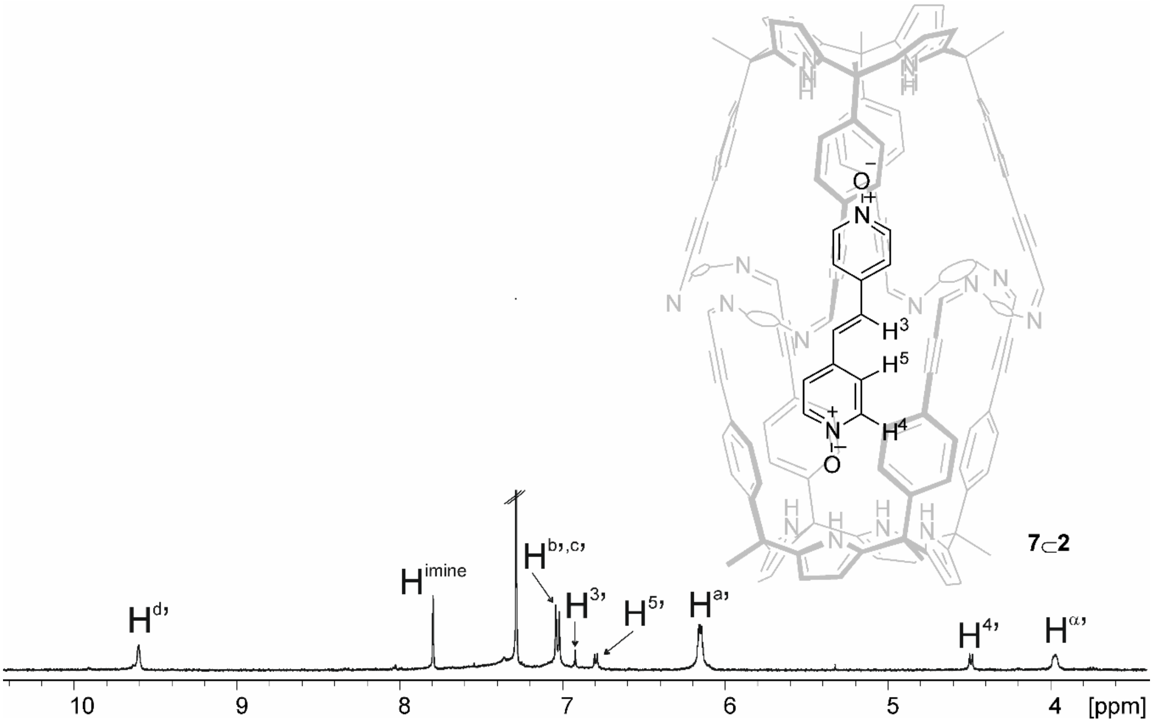

Figure S18. ^{1}H NMR spectrum of a $CDCl_3$ solution of calixpyrrole **3**, 1,2-di(4-pyridyl)ethylene *N*,*N*'-dioxide and (1*R*,2*R*)-(−)-1,2-diaminocyclohexane in a 1:0.5:2 molar ratio. See Figure S1 and Figure S13 for proton assignment. Bound protons marked with primed letters and numbers.

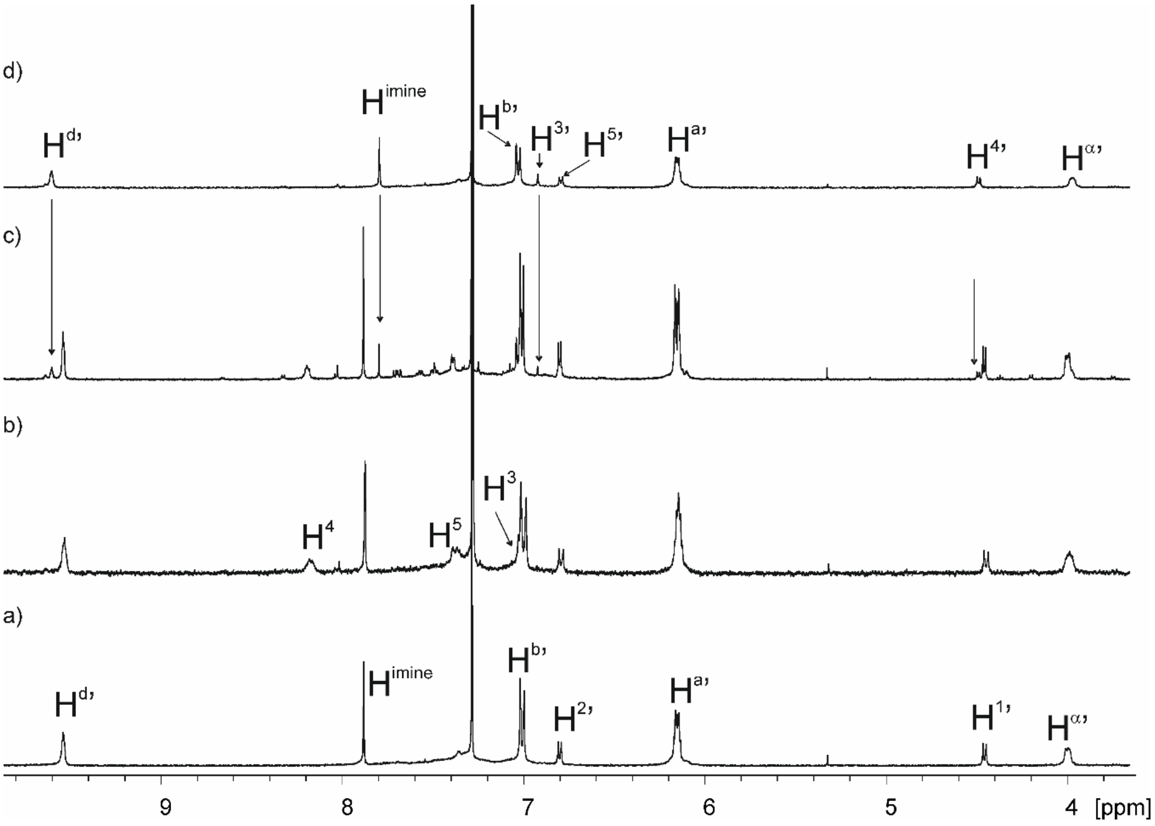

Figure S19. ^{1}H NMR spectra of a) **6**⊂**2** capsule b) **6**⊂**2** capsule and 1 equivalent of guest **7** c) **7**⊂**2** capsule and 1 equivalent of guest **6** after 1 week d) **7**⊂**2** capsule.

4. DOSY NMR experiments

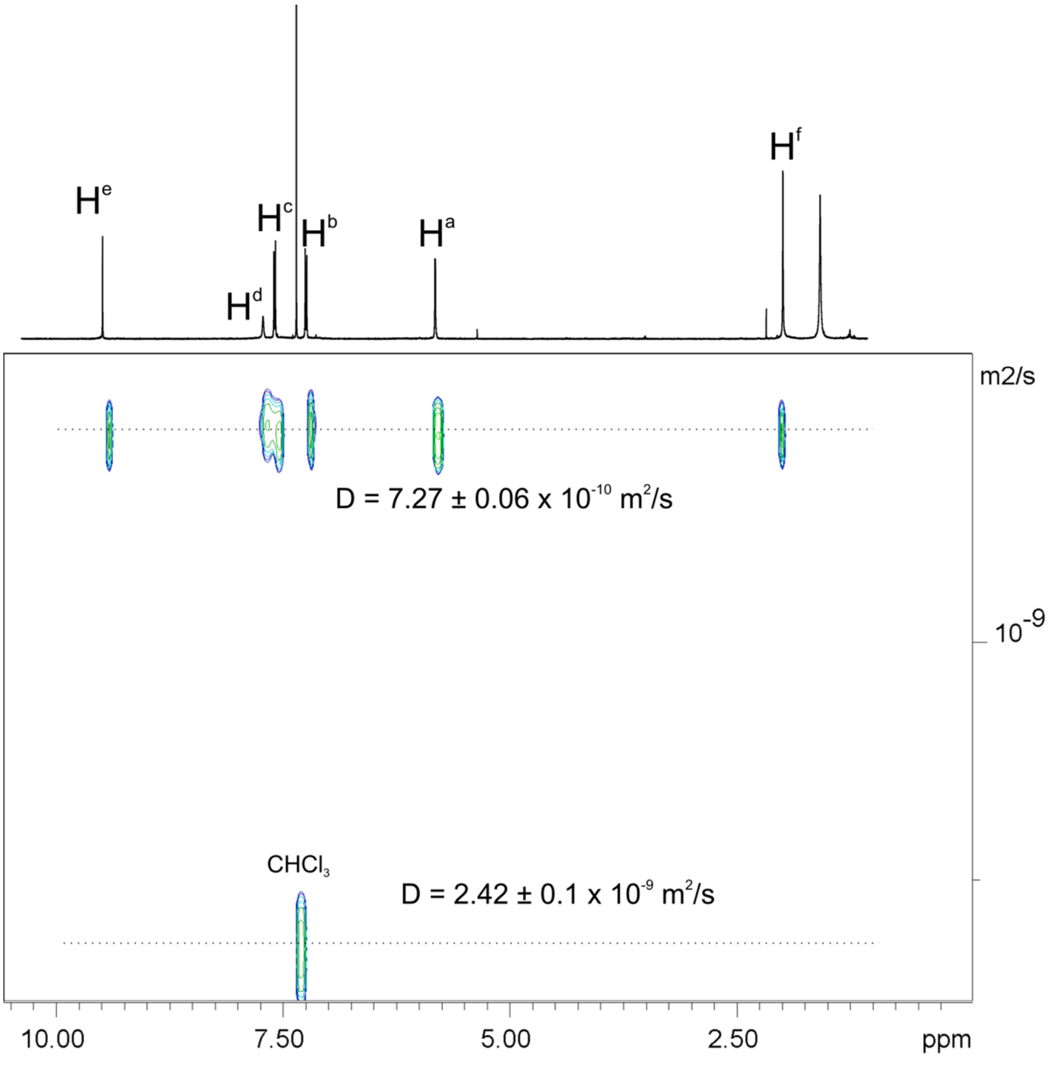

r = 5.5 ± 0.04 Å

Figure S20. ^{1}H pseudo-2D DOSY plot of a $CDCl_3$ solution of calixpyrrole **3**. Data fitted to a monoexponential function. See Figure S1 for proton assignment.

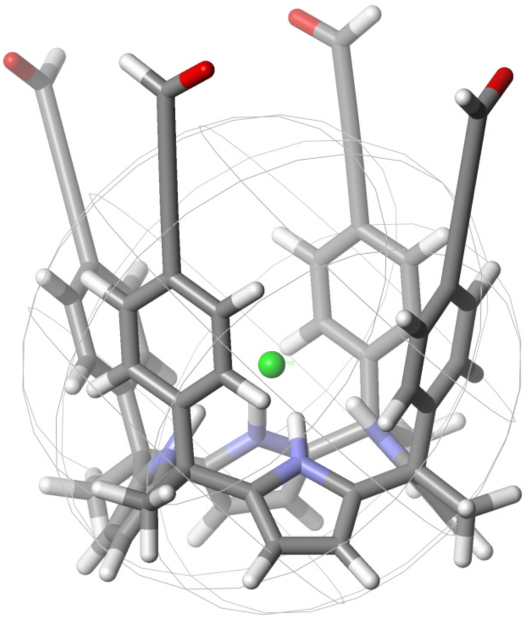

Figure S21. Energy-minimized structure (MM3) of calixpyrrole **3** encircled in a sphere of 5.5 Å radius.

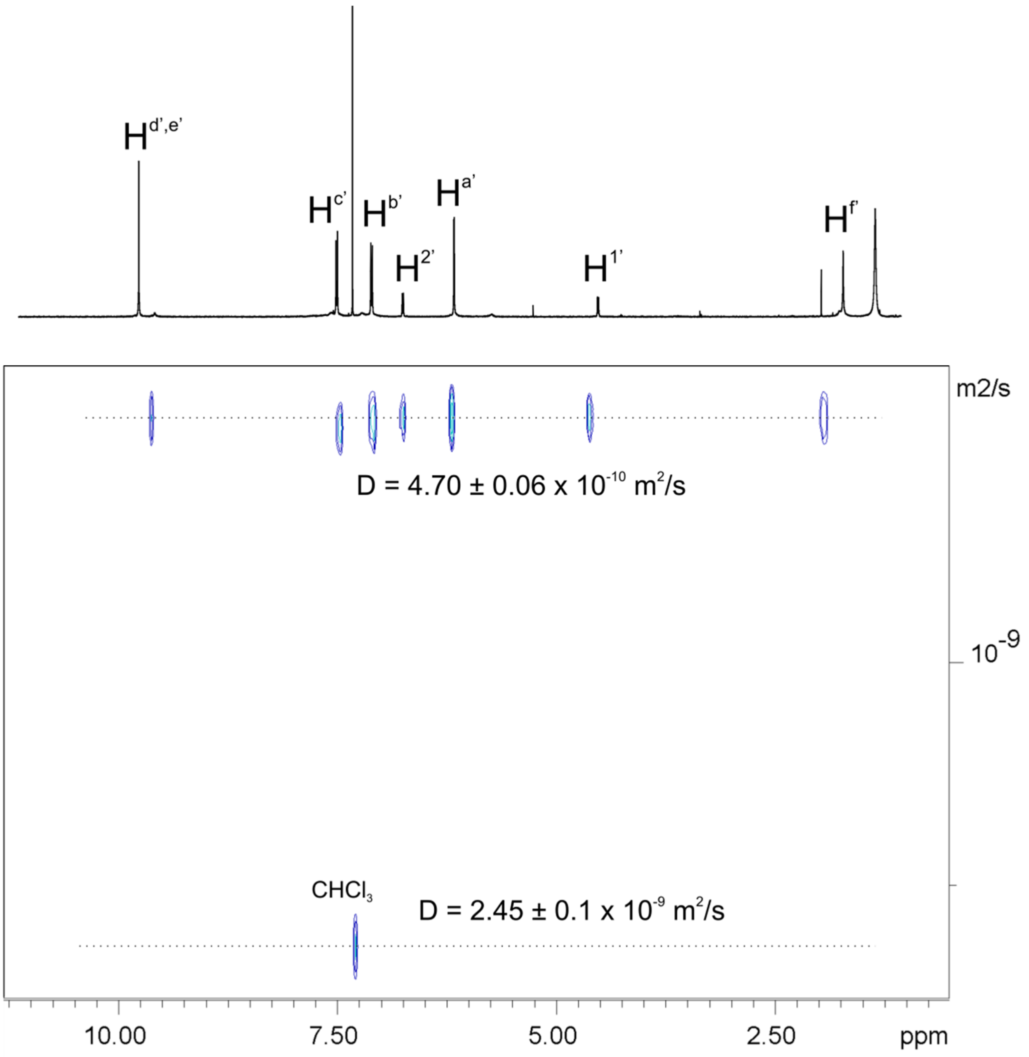

r = 8.5 ± 0.1 Å

Figure S22. ^{1}H pseudo-2D DOSY plot of a $CDCl_3$ solution of calixpyrrole **3** and 1,2-bis(4-pyridyl)acetylene *N*,*N*'-dioxide in a 2:1 molar ratio. Data fitted to a monoexponential function. See Figure S1 and Figure S7 for proton assignment. Hydrodynamic radius calculated with the Stokes-Einstein equation.

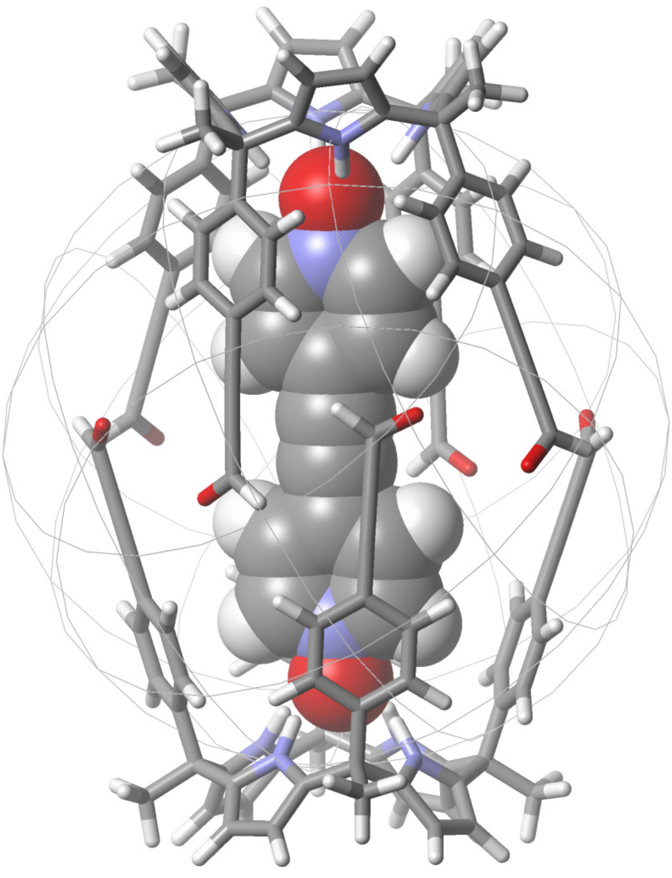

Figure S23. Energy-minimized structure (MM3) of the capsular assembly formed by two units of calixpyrrole **3** and a molecule of 1,2-bis(4-pyridyl)acetylene *N*,*N*'-dioxide encircled in a sphere of 8.5 Å radius.

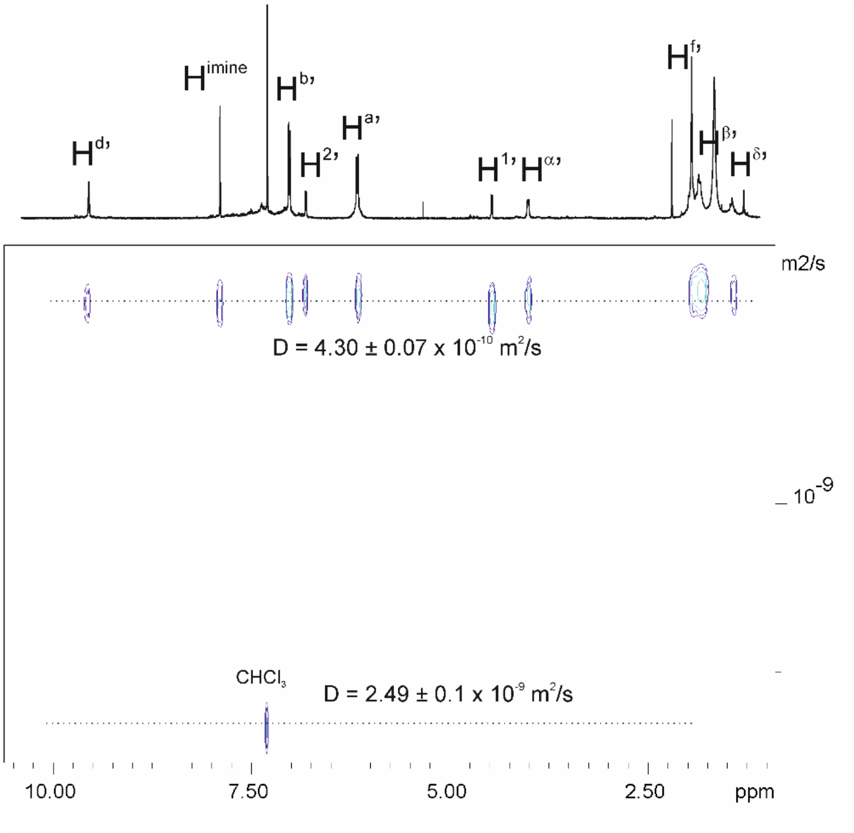

r = 9.4 ± 0.2 Å

Figure S24. ^{1}H pseudo-2D DOSY plot of a $CDCl_3$ solution of calixpyrrole **3**, 1,2-bis(4-pyridyl)acetylene *N*,*N*'-dioxide and (1*R*,2*R*)-(−)-1,2-diaminocyclohexane in a 1:0.5:2 molar ratio. Data fitted to a monoexponential function. See Figure S1, Figure S7 and Figure S13 for proton assignment. Hydrodynamic radius calculated with the Stokes-Einstein equation.

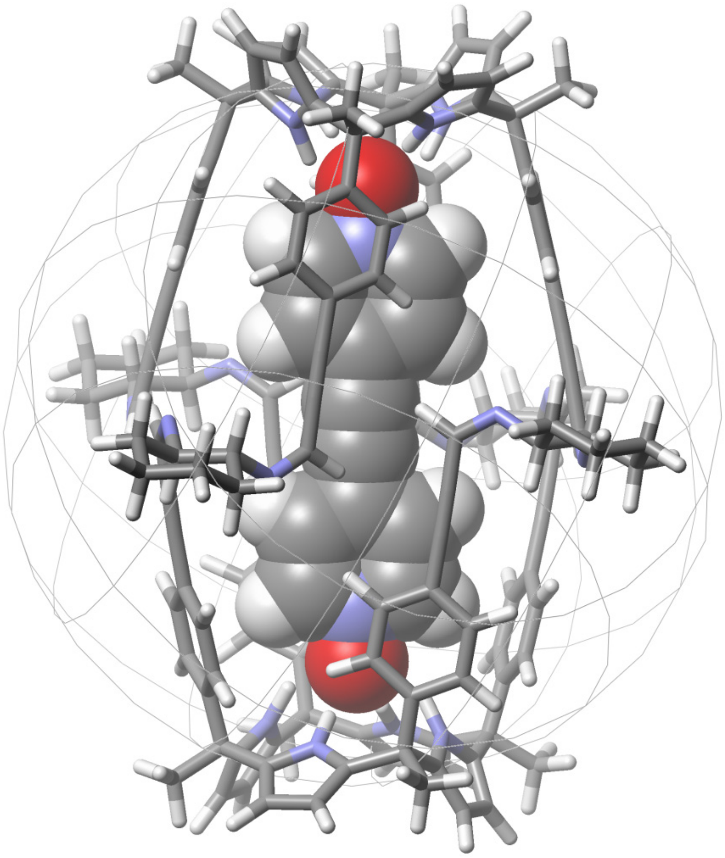

Figure S25. Energy-minimized structure (MM3) of the capsular assembly formed by two units of calixpyrrole **3**, a molecule of 1,2-bis(4-pyridyl)acetylene *N*,*N*'-dioxide and four molecules of (1*R*,2*R*)-(−)-1,2-diaminocyclohexane as linkers encircled in a sphere of 9.4 Å radius.

5. Mass spectrometry experiments

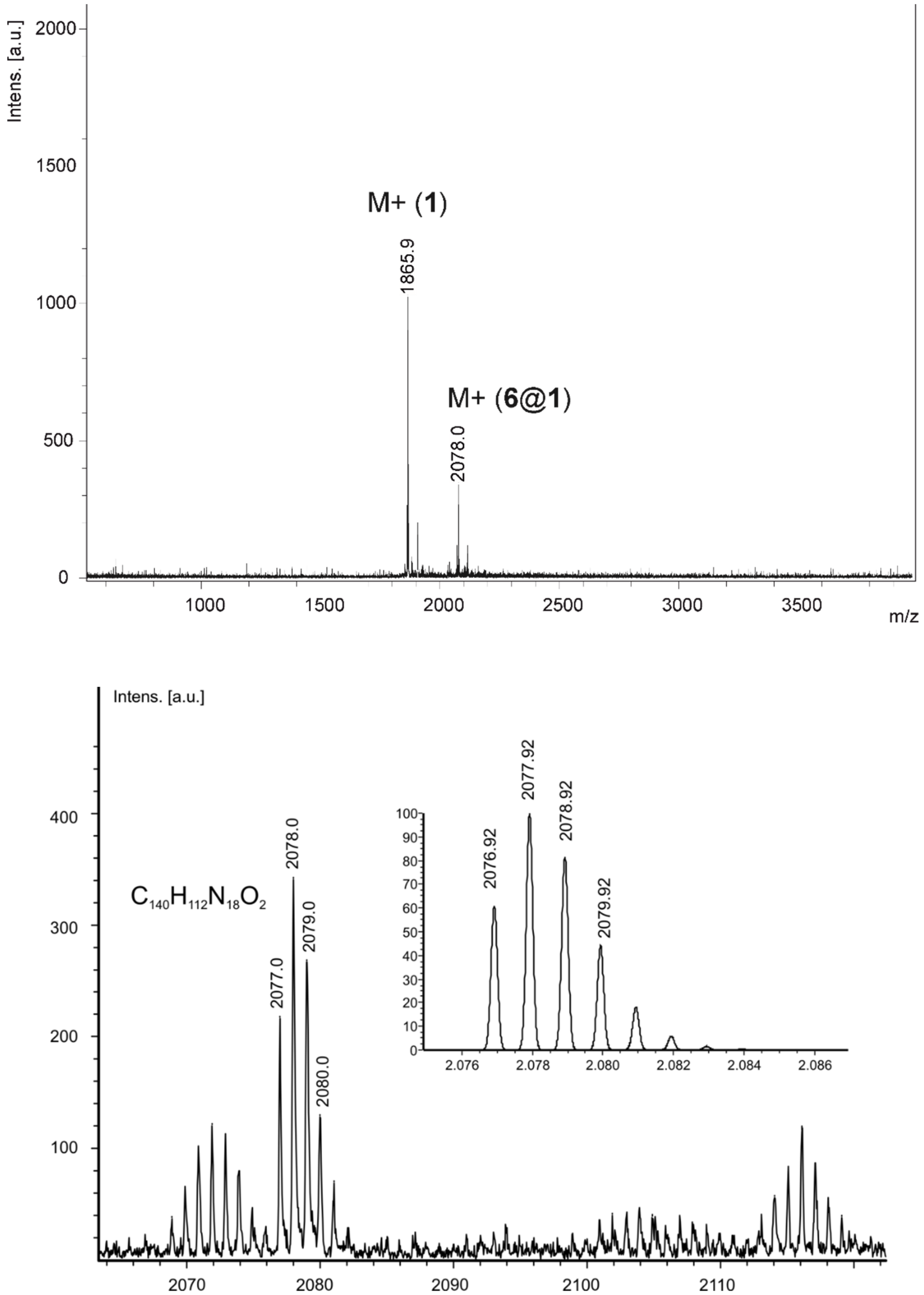

Figure S26. MALDI+ spectra of a $CDCl_3$ solution of calixpyrrole **3**, 1,2-bis(4-pyridyl)acetylene *N*,*N*'-dioxide and ethylenediamine in a 1:0.5:2 molar ratio. Mass calculated for $C_{140}H_{112}N_{18}O_2$ (capsule **6**$\subset$**1**) 2076.92, found 2077.0. Matrix used: DCTB

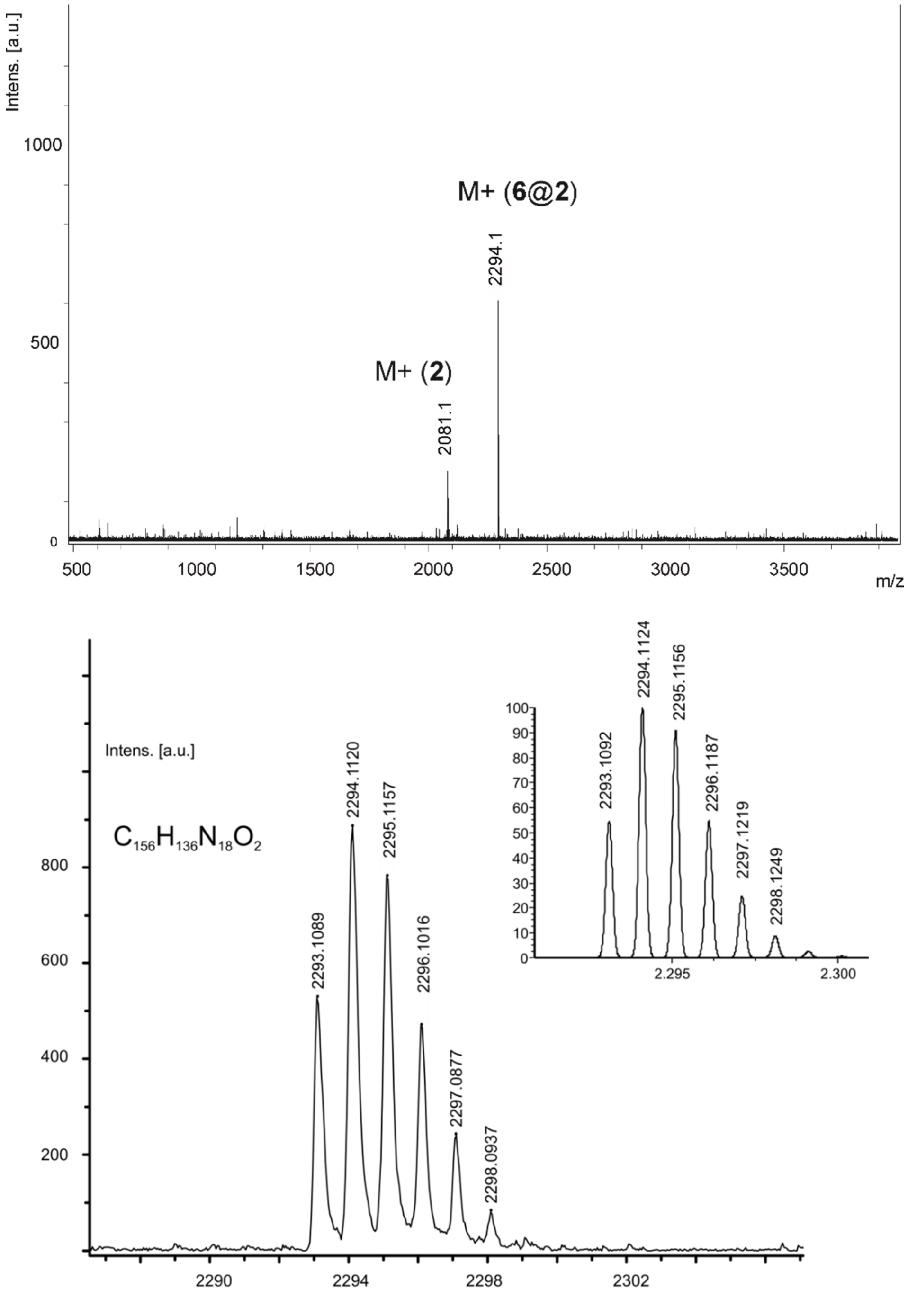

Figure S27. Simulated and experimental high resolution MALDI+ spectrum of a $CDCl_3$ solution of calixpyrrole **3**, 1,2-bis(4-pyridyl)acetylene *N*,*N*'-dioxide and (1*R*,2*R*)-(−)-1,2-diaminocyclohexane in a 1:0.5:2 molar ratio. Mass calculated for $C_{156}H_{136}N_{18}O_2$ (capsule **6**⊂**2**) 2293.1092, found 2293.1089. Matrix used: DCTB

6. Molecular modelling

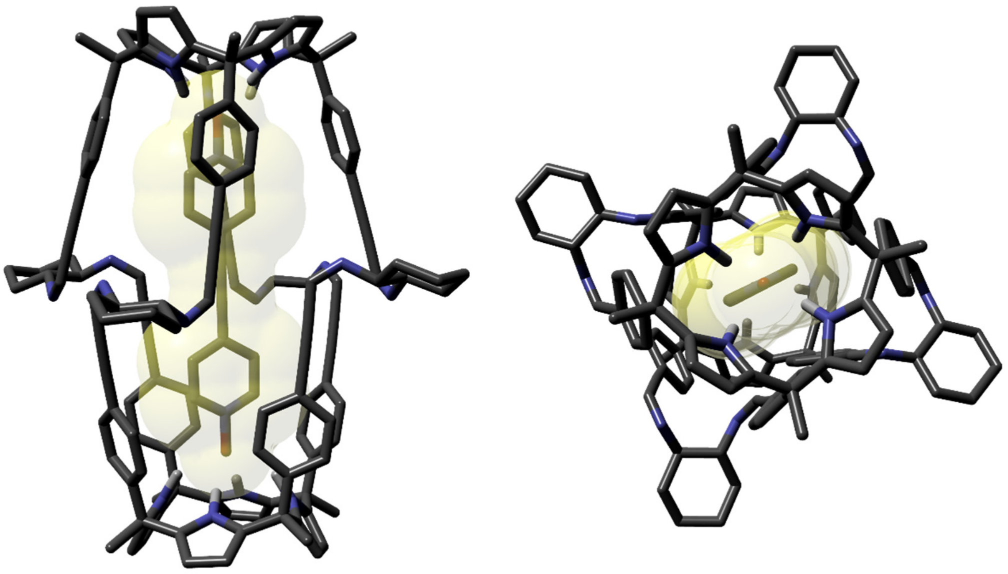

Figure S28. Side and top views of the energy-minimized structure (MM3) of the diastereomeric capsular assembly *P*-(1*R*,2*R*)-**6**$\subset$**2** observed experimentally. The structure agrees perfectly with the structure observed in solution. Non-polar hydrogen atoms removed for clarity. The capsular assembly is depicted in stick representation. Energy = 309.55 kcal/mol.

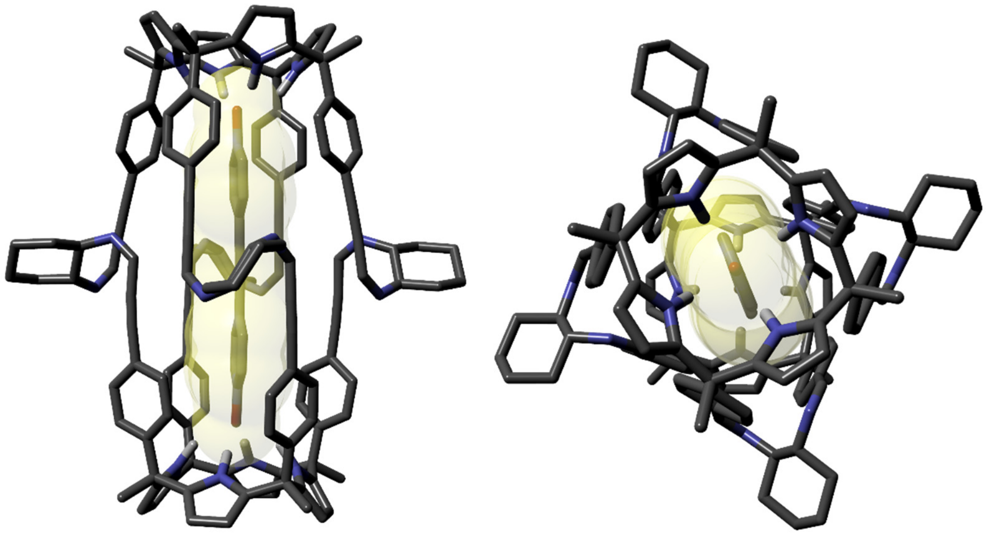

Figure S29. Side and top views of the energy-minimized structure (MM3) of the diastereomeric capsular assembly *M*-(1*R*,2*R*)-**6**$\subset$**2** not detected experimentally. Non-polar hydrogen atoms removed for clarity. The capsular assembly is depicted in stick representation. Energy = 334.28 kcal/mol.
